# Preventing sexual violence in college men: a randomized-controlled trial of *GlobalConsent*

**DOI:** 10.1186/s12889-020-09454-2

**Published:** 2020-09-01

**Authors:** Kathryn M. Yount, Tran Hung Minh, Quach Thu Trang, Yuk Fai Cheong, Irina Bergenfeld, Jessica M. Sales

**Affiliations:** 1 Hubert Department of Global Health and Department of Sociology, Emory University, 1518 Clifton Rd. NE, Atlanta, GA 30322 USA; 2 Center for Creative Initiatives in Health and Population, 48, 251/8 Nguyen Khang str, Cau Giay, Hanoi, Vietnam; 3 Department of Psychology, Emory University, 36 Eagle Row, Atlanta, GA 30322 USA; 4 Department of Behavioral, Social, and Health Education Sciences, Rollins School of Public Health, Emory University, 1518 Clifton Rd NE, Atlanta, GA 30322 USA

**Keywords:** Behavioral change communication, Bystander self-efficacy, Bystander behavior, Campus sexual assault, Educational entertainment (edutainment), Sexual violence, Social cognitive theory, Social norms theory, Vietnam

## Abstract

**Background:**

Sexual violence—any sexual act committed against a person without freely given consent—disproportionately affects women. Women’s first experiences of sexual violence often occur in adolescence. In Asia and the Pacific, 14% of sexually experienced adolescent girls report forced sexual debut. Early prevention with men that integrates a bystander framework is one way to address attitudes and behavior while reducing potential resistance to participation.

**Methods:**

This paper describes a study protocol to adapt RealConsent for use in Vietnam and to test the impact of the adapted program—GlobalConsent—on cognitive/attitudinal/affective mediators, and in turn, on sexual violence perpetration and prosocial bystander behavior. RealConsent is a six-session, web-based educational entertainment program designed to prevent sexual violence perpetration and to enhance prosocial bystander behavior in young men. The program has reduced the incidence of sexual violence among men attending an urban, public university in the Southeastern United States. We used formative qualitative research and the Centers for Disease Control and Prevention’s Map of the Adaptation Process to adapt RealConsent. We conducted semi-structured interviews with college men (*n* = 12) and women (*n* = 9) to understand the social context of sexual violence. We conducted focus group discussions with university men and stakeholders (*n* = 14) to elicit feedback on the original program. From these data, we created scripts in storyboard format of the adapted program. We worked closely with a small group of university men to elicit feedback on the storyboards and to refine them for acceptability and production. We are testing the final program—GlobalConsent—in a randomized controlled trial in heterosexual or bisexual freshmen men 18–24 years attending two universities in Hanoi. We are testing the impact of GlobalConsent (*n* = 400 planned), relative to a health-education attention control condition we developed (n = 400 planned), on cognitive/attitudinal/affective mediators, prosocial bystander behavior, and sexual violence perpetration.

**Discussion:**

This project is the first to test the impact of an adapted, theoretically grounded, web-based educational entertainment program to prevent sexual violence perpetration and to promote prosocial bystander behavior among young men in a middle-income country. If effective, GlobalConsent will have exceptional potential to prevent men’s sexual violence against women globally.

**Trial registration:**

U.S. National Library of Medicine Clinicaltrials.govNCT04147455 on November 1, 2019 (Version 1). Retrospectively registered. Protocol amendments will be submitted to clinicaltrials.gov.

## Background

### Sexual violence is global and disproportionately burdens women

*Sexual violence* is any sexual act committed against a person without freely given consent. Sexual violence ranges from unwanted sexual contact and non-contact sexual experiences to attempted or completed forced penetration [1]. Among sexually experienced adolescent women 15–19 years, reported experiences of forced sexual debut have been common worldwide (14.9%), including in the Asia/Pacific region (13.8%) [2]. The physical, psychological, and economic aftermath of sexual violence often has been severe for its victims [3, 4], totaling $127 billion yearly in medical costs, lost earnings, pain, suffering, and lost quality of life in the US alone [5]. Men also have experienced sexual violence, but women have comprised 91% of victims [6].

### Norms of masculinity and violence in Vietnam

Social cognitive theorists have argued that behavior is learned by observing the behavior of others and its associated rewards or consequences [7, 8]. According to Hearn [9], “any learning (of violence or indeed anything else) is…gendered.” In lower- and middle-income countries (LMICs) [10–14], witnessing male-on-mother violence or experiencing maltreatment in childhood has predicted men’s subsequent perpetration of partner violence. Thus, boys have learned that violence is possible, often is performed by older men, and is enacted in the context of “male domination more generally” (9, p. 27).

In Vietnam, Rydstrøm [15] has characterized “violent inter-generational practices” between grandfathers, fathers, and grandsons or sons as “tangible manifestations of a masculine discourse... composed of a tradition of patrilineal ancestor worship [and] ideas about honor” (pp. 329–330). Confucianism has stressed moral obligations in hierarchical relationships, such as father with child, older brother with younger brother, and husband with wife. Older persons have been respected for their greater proximity to deceased ancestors, and the superior has been expected to educate the inferior, who should return obedience, gratitude, and filial piety [16, 17]. Gendered privilege, such as descent being traced through fathers, has been embedded in this kinship system [18, 19]. As such, senior men have been viewed as superior to senior women [20], and a senior man symbolically and customarily has headed the household [16, 21, 22]. The head of household has had the right to raise, educate, and discipline junior male and female kin [18, 19, 23], for example, by instilling fear or using physical punishment [24, 25]. Such methods have taught children “their subordinate role in the learning process” and the nature of social relations generally [26]. For instance, a mother or grandmother would rarely beat a son, because doing so would challenge the male household head’s authority to assess when corporal discipline is “needed” [15].

Violent intergenerational relations among male kin also have reflected local ideas about “hot” superior masculinity and “cool” inferior femininity [15]. When a father has corporally punished a son, the father has elevated his own masculinity and has demoted the boy to an inferior (feminine) position [15]. Boys may even have described such violence as “justified” if they saw themselves as at fault [15]. In male–male relations in Vietnam, the use of violence to establish hierarchies and to reinforce violence has been one way in which dominant masculinity has been expressed [26].

New household arrangements have challenged these age-gender hierarchies [19, 27–29]. Since the 1990s, schooling attainments have reached gender parity [30] and women [31] increasingly have migrated for work, spurring some husbands to assume unpaid family work [32]. These deviations from customary age-gender hierarchies may have threatened the “entitlements” of some senior men, spurring violence as a means to reinstate male dominance [25]. In this way, men’s dominance and use of violence not only has been embedded in local patrilineal kinship systems, but also may have increased in response to structural changes in gender relations.

### Sexual norms in contemporary Vietnam

In Vietnam, rigid gender norms have contributed to gender inequalities [33, 34] in sexual relations, as well. General expectations of women’s submissiveness, obedience, and passivity and men’s strength, assertiveness, and dominance [35] have been associated with diminished self-efficacy in sexual communication among women in heterosexual relationships [33].

For many years, premarital sex in Vietnam has been viewed as morally corrupt, and women’s sexuality has been a subject of scrutiny [22]. Historically, gender norms associated a woman’s virginity until marriage with her dignity and reputation [34–36] as well as her ‘natural’ role as a mother and builder of a happy family [22]. Having premarital sex promised severe repercussions, including reputational damage to the woman and her family, and other public sanctions [22, 36]. With economic and social liberalization starting in the 1980s, sexual norms began to change radically [37]. Ethnographers have documented trends toward the commodification of sex, particularly among heterosexual men [37]. For young people in urban Vietnam, sex and relationships have taken on a transactional, and sometimes adversarial [38], nature, with women feeling pressure to avoid being taken advantage of by young men interested in sex, but not marriage.

Despite these societal shifts, young women’s virginity has remained a prevalent social norm [34, 35]. Many parents still expect daughters to abstain from premarital sex, emphasizing the reputational damage that daughters and their families could endure if a daughter were to transgress this norm [36, 39]. Other family members and friends have tended to reinforce these messages [34]. Thus, social pressure for girls to be chaste has remained strong, especially outside major metropolitan areas [35].

These changes have contributed to conflicting generational norms about sex in relationships [33, 40]. Alongside persistent family norms of female virginity, women’s peers are reframing premarital sex as normal [40], an expression of love, and a way to strengthen a committed relationship [41]. An emerging norm among young people is that those in relationships who do not have sex do not truly love each other [36]. Premarital sex has become more common [42], and attitudes about premarital sex are shifting, albeit more slowly among women than among men [36, 43]. Thus, young women are facing a conflict between old and new norms [44]. “At a confusing crossroads,” they are trying “to balance the old and new in their concepts of love, the value of virginity and pre-marital and extra-marital sexual relations” [45].

### The legal framework in Vietnam favors sexual violence prevention

In the context of these conflicting sexual norms, the State has pursued legal reforms to promote gender equality and to prevent gender-based violence. The Law on Marriage and Family in 1986 (updated in 2000) gave men and women equal rights in marriage, and the Law on Gender Equality in 2006 sought to eliminate gender discrimination and to curb differential rights based on gender [46, 47]. The Penal Code of 1989 (updated in 1999, 2015, and 2017) defined penalties for acts of sexual violence [48, 49]. Rape was defined legally as an act committed by someone who, through violence or its threat, or by using the victim’s helplessness or other means, forces the victim to have sexual intercourse against her will. Rape carries a punishment of imprisonment for two to seven years. Convicted offenders could be barred from certain jobs or positions of responsibility for one to five years. Terms of up to 20 years or even life imprisonment or death could be imposed when victims suffer grievous bodily harm, perpetrators are known HIV carriers, or victims die or commit suicide because of the rape [50]. Death could be the punishment if the victim is under age 16 and the offense causes 61% or more whole personal impairment. A Resolution dated 1 Oct 2019 provides guidance on the application of several regulations of Articles 141–147 of the Criminal Code and resolutions to difficulties encountered during settlement of cases of sexual exploitation and abuse of persons under age 18. A Law on Domestic Violence in 2007 (and updated in 2017) codified definitions of physical, psychological, sexual, and economic violence as a step to prevent these forms of violence [51, 52].

### Preventing sexual violence must engage men

Despite legal reform, sexual and other forms of violence against women have persisted in Vietnam [47, 52]. In our research outside Hanoi, men have discounted, excused, or denied acts of violence [53]. As a result, men’s reported rates of sexual violence perpetration (0.2%) have been lower than women’s reported rates of victimization (12.0%) [13]. Thus, prevention with men has been considered crucial to create an environment where women’s bodily integrity and freedom from violence are possible [54]. Yet, young men have been difficult to reach [55, 56] and have resisted programs that target sexual violence, not identifying themselves as potential perpetrators [57]. Limited data also have suggested that the behavior of bystanders, or witnesses of sexual violence, is gender-specific [58]. Although evidence from rigorous evaluations has been limited [59], prevention with men that integrates a bystander framework may address attitudes and behaviors while decreasing resistance to participation [60–62] because men are treated as women’s “allies” [63].

### Innovation and contribution

This project disrupts several paradigms related to the prevention of sexual violence. First, interventions to prevent sexual violence by young men have been rare in LMICs [64]. This project is the first to adapt RealConsent and to test the impact of the adapted program (GlobalConsent) on sexual violence prevention and prosocial bystander behavior in college-going men in a middle-income country. Second, even in higher-income countries, most programs have entailed in-person, small-group formats, with limited reach, standardization, and impact [65, 66]. RealConsent is a theory-driven, evidence-based, educational entertainment program tailored to young men and delivered in six episodes via the web. Each of these programmatic features of RealConsent warrant comment:

1. **Gender-specific programming.** Gender-specific content is more effective than gender-generic content in behavioral interventions to reduce drinking [67] and to prevent rape [68]. As such, programs to prevent sexual violence perpetration should be gender-specific [69, 70]. RealConsent is the only gender-specific program commercially available for cross-cultural adaptation.
2. **Evidence-based approach**. RealConsent integrates well-known behavioral change techniques, such as providing information and instruction on obtaining effective consent for sex and intervening safely, modeling communication and intervening behaviors, and showing positive outcomes for getting consent and intervening plus negative outcomes for perpetrating and not intervening, and reinforcing with support and positive feedback [71].
3. **Interactive, problem-based learning via educational entertainment**. RealConsent uses several best practices, such as didactic presentation of material via video and infographics, problem-based learning with interactivity, and short videos or animations to model behavior. Problem-based learning activities with real-time feedback are core elements. These activities require the user to make a decision (e.g., “do something” or “stay out of it”) after viewing a brief filmed scenario. Based on his decision, the user is guided through a sequence of outcomes so each consequence of a poor choice is shown. This feature is based on theories of social learning by reinforced practice, and this formula is enhanced by adding educational entertainment, starting and ending each module with a three-minute episode of a professionally produced serial drama based on formative research. In the US, most male participants rated the episodes as an *effective* or *highly effective* way to learn about consent and prosocial intervening behaviors [72].
4. **Web-based and mobile website platforms**. Behavioral change techniques initially were developed for in-person delivery to small groups. Today, researchers are applying best practices for behavioral change to web and mobile applications. Online behavioral-change programs have tended to have larger effects when they have included more behavioral change techniques [73], have delivered content that resonates with the user [74], and have integrated audio, graphics, and interactivity [75]. For example, a gender- or ethnicity-specific vignette may have stronger cognitive or motivational effects, because personal relevance enhances learning and increases motivation to complete the program.

The high video-production quality of RealConsent, with rigorous scientific content based on principles of behavioral change tailored to young men, will appeal to leaders who seek to reduce sexual violence at their educational institutions. RealConsent has reduced the incidence of sexual violence perpetration in men attending an urban, public university in the Southeastern US [76]. Once developed, web-based programs like RealConsent can be less resource-intensive to adapt [76–78]. These techniques, and the high-quality production format, have been retained to the extent possible in GlobalConsent (results of the adaptation to be presented elsewhere). If found to be effective, GlobalConsent has exceptional promise to be a cost-effective means to reduce the incidence of sexual violence against women globally.

## Methods/design

### Integrated theory of change

**Fig. 1:**
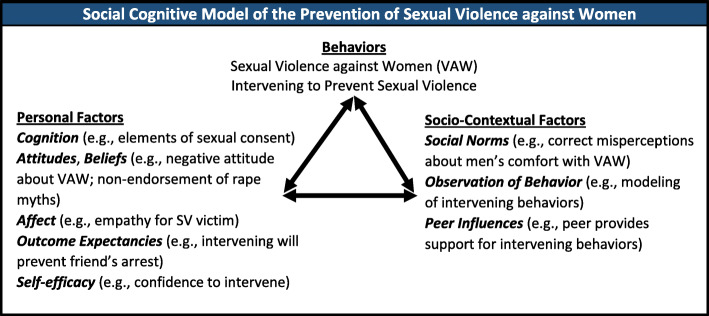
Social Cognitive Model of the Prevention of Sexual Violence against Women

RealConsent was developed based on formative research and three synergistic theoretical frameworks: social cognitive theory [79], social norms theory [80], and the bystander education model [81]. Social cognitive theory, the overarching framework for RealConsent, posits that behavior is a function of the interplay of socio-contextual factors, personal factors, and behavior (Fig. 1). Applied to violence against women, social cognitive theory posits that the broader context of social norms, social support, and the media puts men at risk for perpetrating sexual violence (and women at risk for experiencing it). Social norms theory posits that social expectations about a behavior, such as sexual violence, influence that behavior based on people’s perceptions–or misperceptions–of those expectations. Personal factors, such as cognitions, attitudes, affect, and biological events, interact with contextual factors and behavior. Bystander education suggests that personal and behavioral changes related to intervening in a situation of potential sexual violence could, in turn, affect social norms about sexual violence.

Based on these theories, RealConsent has integrated content to achieve two behavioral goals by changing seven theoretically and empirically derived cognitive, attitudinal, and affective mediators. The two goals are: 1) to increase prosocial intervening behaviors, such as trying to stop a peer who is being coercive, that reduce the risk for sexual violence perpetration, and 2) to prevent sexually violent behavior toward women. The seven mediators include 1) increasing knowledge about the elements of sexual consent, 2) increasing knowledge and skills to intervene safely, 3) correcting misperceptions in norms about sexual violence and rape, 4) reducing negative attitudes about date rape, 5) promoting positive masculinity, 6) enhancing skills in sexual communication, and 7) increasing empathy for the victims of sexual violence. Thus, the content of RealConsent has drawn on social-cognitive theory and social-norms theory and maps onto an integrated theory of change targeting seven mediators to change bystander behavior and sexually violent behavior.

### Evidence of efficacy of RealConsent

To evaluate the program and underlying theory of change, a random probability sample of 743 male undergraduate students (18–24 years) attending a large, public, urban university in the southeastern United States was recruited online and randomized to RealConsent (*n* = 376) or a Web-based general health education attention-control program (*n* = 367) [76]. This age group was a useful one for sexual violence prevention for several reasons. First, adolescence is a pivotal period for the onset of sexual violence perpetration, and the median age at first perpetration of various forms of sexual violence has been documented to be 16–17 years [82]. Also, in non-Western settings, universities are key entry-points for efforts to prevent sexual violence, because women increasingly are entering higher education, are exposed to the risk of sexual violence as university students [83, 84], and face substantial adverse consequences without institutional policies or response programming.

Participants in the original RealConsent trial were surveyed online at baseline, post-intervention, and 6-months post-intervention. Six 30-min media-based and interactive modules covering knowledge of informed consent, sexual communication skills, the role of alcohol and male socialization in sexual violence, empathy for rape victims, and bystander education were delivered via a password-protected Web portal. At 6-month follow-up, compared to the control group, the RealConsent group intervened more often and engaged in less sexual violence (*p* < .05). They reported greater knowledge about the legal definitions of sexual assault and the elements of effective consent (*p* < .001). They reported lower adherence to rape myths, negative date-rape attitudes, hyper-gender ideology, and hostility to women (*p* ≤ .01). They reported greater empathy for rape victims (*p* < .001), greater intentions to intervene (*p* = .04), less positive outcome expectancies for nonconsensual sex (*p* = .03), more positive outcome expectancies for intervening (*p* < .001), and less comfort with other men’s inappropriate behavior (*p* < .001). Thus, results supported the efficacy of RealConsent, its underlying theory of change, and potential for adaptation.

### Setting and study sites

The undergraduate system in Vietnam was considered a suitable context for adapting RealConsent, given important similarities with the US undergraduate system. In Vietnam, undergraduate study is 4–6 years, with two foundational years and 2–4 years for specialization. Except for political education and national defense, universities design their own curricular and extra-curricular activities and maintain networks through the Ministry of Education and Training (MoET), professional associations, and Youth Union, all potential pathways to bring GlobalConsent to scale in the future.

The sites selected for this project were Hanoi Medical University (HMU) and Thang Long University (TLU), both in Hanoi. HMU is a 100-year-old state school with dental, medical, nursing, nutrition, public health, and paramedical/ophthalmology technical programs. Its 1000 enrolled students are 40% men. TLU is a 24-year-old private university with 15 departments ranging from math to business administration and the social sciences. Its 7000 enrolled students are 45–50% men. Both schools provided letters of support for adaptation of RealConsent with their students.

### Overview of study phases

This project has used a mixed-methods design and the Centers for Disease Control and Prevention (CDC) *Map of the Adaptation Process* (MAP): *A Systematic Approach for Adapting Evidence-Based Behavioral Interventions* [85] to adapt RealConsent for use with college men in Vietnam and to test whether the adapted program (GlobalConsent): **H1** improves men’s cognition, attitudes, and affect regarding sexual violence against women; **H2** increases their tendency to intervene to prevent such violence; and **H3** reduces their propensity to perpetrate sexual violence against women. We have been interested to understand through which pathways the adapted program may achieve its effects.

### MAP framework to adapt RealConsent for Vietnam

Considerable work on adapting evidence-based interventions (EBIs) has drawn on Roger’s [86] theory of diffusion. Accordingly, adaptation is the degree to which a user modifies an innovation, while retaining its core elements, in the process of its adoption and implementation in a new context. The CDC developed five sequential steps of MAP to document these modifications while adapting EBIs to new populations and contexts [85]. Step 1, ***assess***, involves assessing the target population, the EBI being considered for implementation, and the implementing agency’s capacity to employ the intervention. Step 2, ***select***, entails determining whether to adopt the EBI without adaptations, to implement the EBI with adaptation, or to choose another EBI and to start the MAP process over. Step 3, ***prepare***, involves making the needed adaptations to the EBI while retaining its core elements. Step 4, ***pilot***, entails pilot testing the adapted intervention and developing a plan for implementation. Step 5, ***implement***, involves conducting the entire adapted intervention in a large enough sample over a sufficient follow-up period to test its efficacy on primary and secondary outcomes. This process helps to ensure that a selected EBI is suitable for the target population, the adapted EBI is relevant for the target population and preserves core elements of the original EBI, the adapted EBI is within the agency’s capacity to deliver, the adaptation process is documented, and impacts of the adapted EBI on primary and secondary outcomes are tested in the setting of interest.

To implement the MAP process, we have undertaken the project in two phases**.** In Phase I, we conducted formative qualitative research to implement Steps 1–4 of the MAP process (assess, select, prepare, and pilot) [87]. In Phase II we have been conducting a randomized controlled trial (RCT) to test the impact of the adapted program (GlobalConsent), versus a health-education attention-control condition, on preventing sexual violence and enhancing prosocial bystander behavior in freshmen college men at two universities in Vietnam. The RCT is on-going, and study results are forthcoming. Here, we have described the study protocol.

### Phase I: formative qualitative research to adapt RealConsent

Formative qualitative research is an activity conducted at the start of the Social Behavior Change Communication (SBCC) project design process [88]. Such research is essential to design program materials, tools, and approaches that are well suited to the local context. In this study, formative qualitative research provided an in-depth account of the local context to which RealConsent was to be adapted [89, 90], revealing norms, attitudes, and strategies [91, 92] requiring consideration to ensure relevance, acceptability, and sustainability of the adapted version in this setting [93, 94]. Interviews with men and women undergraduates allowed us to assess the context of sexual and dating relationships among university students and to develop a profile of our target population of college-going men, including personal and socio-contextual risk factors (MAP Step 1). Also, groups of college men and university stakeholders offered feedback on the six original RealConsent modules, allowing us to assess their needs, willingness to collaborate, and perceptions of the original program (MAP Steps 1–2). This assessment enabled us to identify aspects of RealConsent that we could maintain, that required adaptation, and that needed to be removed. Keeping records of the adaptation process, Emory and CCIHP partners adapted the script, and created and assembled new storyboards for the adapted program (MAP Step 3). We pretested these storyboards with a small sample of university men and used their feedback to refine the adapted script and materials until they were acceptable to this group (MAP Step 4). Using these data, we produced a web-based version of GlobalConsent that was designed for delivery on smartphones.

#### Sample eligibility and recruitment

Qualitative research participants were men and women undergraduates and university stakeholders (administrators and faculty) at HMU and TLU. Eligible students were consenting, enrolled men and women, 18 years or older, and ever in a dating relationship. To recruit students, CCIHP personnel contacted department chairs to discuss the study. With departmental agreement, CCIHP talked with faculty about the study and asked them to announce it in their classes, using a flyer with a brief, standard project description that included contact details of the CCIHP study team. Interested students contacted the research team to take part. Researchers at CCIHP sent an informed consent form in advance to all eligible participants, and oral consent was obtained before beginning each interview. CCIHP key personnel held separate meetings with university stakeholders to invite their participation. Eligible participants were recruited until even numbers of men and women students across study universities were represented in the qualitative samples, to ensure saturation of salient themes in the analysis (Table 1).

**Table 1:** Qualitative Data Collection Methods and Samples

		Total interviews/ discussions	Total participants	Year of birth			Currently in dating relationship		
				1998–99	1996–97	1994–95	Yes	No	NA
SSIs Men students	TLU	6	6	2	4	0	5	1	0
	HMU	6	6	3	2	1	3	2	1
SSIs Women students	TLU	5	5	0	5	0	5	0	0
	HMU	4	4	0	4	0	1	3	0
FGDs Men students (lecturers)	TLU	6 (1)	34 (6)	17	13	4	6	17	11
	HMU	6 (1)	35 (6)	27	7	1	19	16	0

Notes. FGD = focus group discussion; HMU=Hanoi Medical University; NA = not applicable SSI=Semi-structured interview; TLU = Thang Long University

#### Data collection methods

In part 1 of the qualitative research, we conducted semi-structured interviews (SSIs) with undergraduate men and women and focus group discussions (FGDs) with undergraduate men and university stakeholders. Semi-structured lists of open-ended questions guided each SSI and FGD, with options to probe unexpected responses (Supplementary File 1. Semi-Structured Interviews and Focus Group Discussion Guides). Interviewers kept field diaries to record their observations and interactions during data collection. Participants were reimbursed ($5 per student $15 per university stakeholder) for their time and received refreshments. Table 1 shows the qualitative data collection methods and associated samples for part 1 of the qualitative work.

In part 2 of the qualitative research, CCIHP recruited nine male students from the two study universities to work intensively with the study team to refine the content of the adapted modules of GlobalConsent and to develop the modules of the attention control program. These nine students continued to work with the study team during the production of both programs.

##### Semi-structured interviews

SSIs allowed us to explore the context of sexual relations and sexual violence among undergraduates in Vietnam (MAP Step 1). The study team developed two SSI guides—one for men and one for women. Question sets covered the following major topics: normative expectations of men and women, sexual relationships between university men and women, relationship experiences and expectations, and perceptions of sexual violence. The guides were developed in English, translated into Vietnamese, and back-translated into English to ensure comparability of meaning. Narratives permitted exploration of how a person’s memories of the past and expectations of the future shaped their understanding of personal experience [95]. They also permitted exploration of how participants perceived acts of sexual violence and other forms of dating violence and understood the concepts of “consent,” “rape,” local laws on sexual and other forms of dating violence, and forms of recourse for survivors. Narratives clarified the context in which, and extent to which, RealConsent required adaptation. Real scenarios and text segments from participants’ narratives were extracted and edited to form the script of the adapted GlobalConsent program.

##### Focus group discussions

Interactions among participants in FGDs can elicit diverse views on a topic, discussion and debate, explanations of issues, and “normative” data that would not emerge from SSIs [96]. We conducted 14 FGDs, distributed evenly across universities (7:7), undergraduate men (*n* = 12), and university staff (*n* = 2) (Table 1). Groups met in an accessible location (e.g., school building). Groups viewed specific modules of RealConsent, with subtitles in Vietnamese, and provided feedback. These data informed decisions about further adaptations of RealConsent, while ensuring that core elements of RealConsent remained or revising the theory of change and associated program content to ensure acceptability (MAP Steps 2, 4).

##### Assessment of GlobalConsent’s acceptability

In MAP step 4, we invited nine male students from the two study universities to review, comment on, and refine all of the program scripts, training content, and presentation format, to ensure attention to the local context and students’ learning styles. These students also tested the platform on which the GlobalConsent and AHEAD training programs were being hosted. They worked intensively with the local research team over 30–35 days for this acceptability assessment, final adaptations, and production.

#### Ethical approvals and informed consent

All data have been and will continue to be collected in accordance with established ethical guidelines for research on gender-based violence, including review and approval by the Institutional Review Board of Emory University (IRB00099860) and the Hanoi University of Public Health (017–384/DD-YTCC). Oral consent forms for adults were developed for each type of interview (e.g., SSI, FGD, survey). The oral consent form covered the following topics: introduction of interviewer and institution s/he represents; purpose of the study: study procedures; anticipated risks or discomfort; benefits; confidentiality; right to refuse or withdraw from the study; contact details of the study coordinators for any questions or concerns; contact details for Emory and CCIHP for any complaints about treatment during the study.

#### Training and fieldwork

Interviewers for the qualitative research were two Vietnamese women and one Vietnamese man who were trained and experienced in conducting interviews on gender-based violence. Training included a focus on ethics and safety when conducting SSIs and FGDs and managing data on sensitive topics, such as sexuality and sexual violence. Among discussed topics were “safe name” for study introduction, obtaining individual consent, maintaining confidentiality, physical safety of participants and researchers, avoiding harmful publicity, and provision of crisis intervention. Researchers discussed with trainees scenarios to prepare for clear, oral informed consent and to maintain confidentiality in data management.

Fieldwork at HMU and TLU occurred concurrently so learning from one site could inform data collection at the other. CCIHP personnel visited both sites regularly to supervise the work. Interviews were conducted in Vietnamese and were digitally recorded. Collected data included audio recordings from the SSIs and FGDs and text from interviewer field diaries (Table 1).

#### Quality control

Vietnamese interviews and group discussions were transcribed verbatim at CCIHP. To maintain confidentiality, each participant was assigned a unique label, and identifying information was removed from the transcripts. Researchers at CCIHP reviewed the transcriptions for quality, accuracy, and confidentiality before translating them into English. Transcripts were uploaded and stored on a HIPAA-compliant, secure network drive maintained by Emory University. Transcribed interviews and group discussions were translated into English. The study team randomly spot-checked the translations to ensure accuracy and fidelity to the Vietnamese transcripts. One additional FGD each with undergraduate men and university staff will be conducted near the end of the analysis to share the findings and to ensure their credibility [97].

#### Data analysis

Qualitative data analysis entails a “search for patterns in data and for ideas that help explain why those patterns are [present]” [98]. The team is combining a grounded-theory approach with narrative analysis and triangulation. A grounded-theory approach entails techniques to identify categories and concepts that emerge from text and to link them into substantive and formal theories [98]. This approach combines deductive and inductive techniques to discern general themes while allowing new understandings and sub-themes to emerge. Narrative analysis preserves the particularities of a narrative while framing individual narratives within larger socio-cultural patterns [99]. Triangulation involves the comparison of data from multiple sources to enhance the validity of data obtained from each source [96]. Thus, notes from field diaries and verbatim transcripts of the FGDs and SSIs have been coded similarly and are being analyzed through constant comparative analysis [100].

Three Emory researchers have familiarized themselves with the data through in-depth readings of all transcripts. These researchers developed an initial codebook that included deductive codes derived from the interview guide, such as sexual violence, consent, and gender norms, as well as inductive codes capturing ideas that emerged from the data, such as sexual intimacy and healthy communication. Initial codes were applied to segments of the data and iteratively refined based on team discussions. Researchers applied the refined codes to two transcripts (representing both genders) to ensure inter-coder reliability. Debriefing about the coding of one transcript allowed the team to refine and clarify the coding system furter. Final revisions were made, and a second round of inter-coder reliability testing resulted in a kappa score of 0.65. A core analytic team reviewed fully coded transcripts to identify potential themes, which are being validated, refined, and contextualized through discussions with the larger team.

Two research assistants coded all notes and English transcripts separately using the MaxQDA qualitative software program. Emory and CCIHP key personnel have met weekly to supervise the coding and have referred to the Vietnamese transcripts, as needed, to verify meaning and to ensure that findings are grounded in the data. The team has discussed cases when the RAs have disagreed, and themes on which two or more team members have agreed are being included in on-going qualitative analyses, for submission for publication separately.

### Phase II impact evaluation of GlobalConsent

#### Study design

**Fig. 2:**
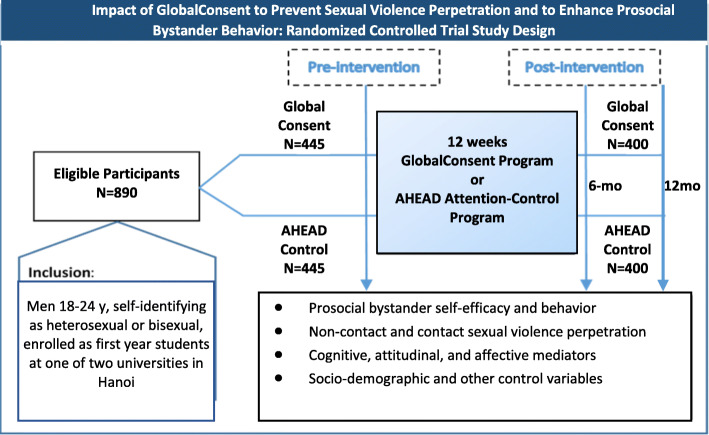
Impact of GlobalConsent to Prevent Sexual Violence Perpetration and to Enhance Prosocial Bystander Behavior: Randomized Controlled Trial Study Design

In line with the adapted theory of change (Fig. 1), and based on learning from the qualitative research, we have been conducting a randomized controlled trial (RCT) with eligible college men to test the impact of the adapted program—GlobalConsent—on promoting prosocial bystander behavior and preventing sexual violence perpetration, through improvements in cognitive, attitudinal, and affective mediators over a 12-month study period, with a baseline survey and two follow-up surveys 6 and 12 months post-baseline (MAP Step 5). Figure 2 depicts the RCT design.

#### Eligible sample and power

Men 18–24 years and self-identifying as heterosexual or bisexual and enrolled at HMU and TLU as freshmen on 9/1/2019 were eligible. According to recent enrollment figures, about 890 men matriculate yearly at HMU and TLU. Assuming a cooperation rate of 90% based on prior studies of this team in Vietnam [12], this pool was considered sufficient to recruit a sample of 800 freshman men for participation in this study.

**Fig. 3:**
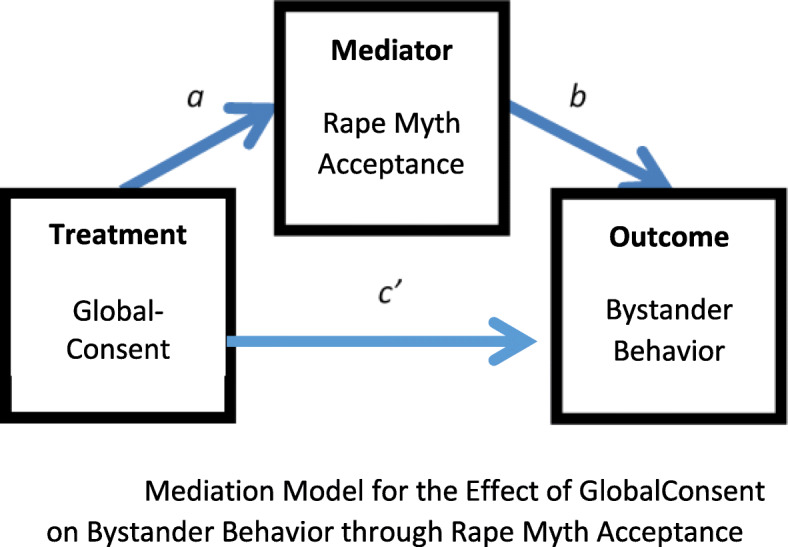
Mediation Model for the Effect of GlobalConsent on Bystander Behavior through Rape Myth Acceptance

We computed the required sample sizes to detect our hypothesized effects with a power of .80. Figure 3 depicts how the effect of GlobalConsent on one of the outcome variables, bystander behavior, is transmitted through one of the mediator variables, Rape Myth Acceptance [101, 102]. In the model, *a* represents the causal pathway between the GlobalConsent treatment and the Bystander Behavior Scale, and the product *ab* captures the indirect effect of GlobalConsent on Bystander Behavior through Rape Myth Acceptance. We used a Monte Carlo approach [103] to calculate the required sample sizes assuming 1) an attrition rate of 10%; 2) Cohen’s small-to-large-effect-categorization [104] regarding the proportion of the variance accounted for, *R*^*2*^, by the model (.02, .13 and .26) using specific values for *a* and *b* [105–107]; and 3) a desired power level of .80. The computer program, Mplus [108], was used to complete the computations. Results of the simulation suggested that our final sample size of 800 had adequate power to detect small, medium and large *a* and *b*, and *ab* for the mediation model (Table 2).

**Table 2:** Sample Size Required to Detect a Mediated Effect with Power of .80 in the Mediation Model with GlobalConsent Treatment

	path *b*		
path *a*	Small (.14)	medium (.36)	large (.51)
Small (.28)	750	450	430
medium (.72)	510	120	90
large (1.02)	490	95	60

The two sizes of paths *a* and *b* are based on Thoemmes et al. (105)

#### Participant recruitment and consent

From the list of all students who matriculated at HMU and TLU on 9/1/2019, there were 362 male students in HMU and 735 male students in TLU. All of these students were invited to participate in the study. At HMU, we invited all male students to attend one orientation meeting to introduce the intervention and the study. Sixteen students did not attend this meeting. At TLU, we organized several meetings with male students based on the class time of different departments during orientation week at the university. There, 189 male students from TLU did not attend these meetings. Seven students, all from TLU, refused to participate in the intervention and surveys after the introduction. The intervention study was introduced as an extra learning curriculum developed under a collaboration between Emory University, TLU, and HMU and having six online modules and pre- and post-program assessments. The students were informed that they would receive about $5 for completing each survey and about $2.5 for completing each learning module. Students who agreed to participate were invited to different sessions organized in a meeting hall or classes where they could complete a tablet-assisted informed consent process and self-interview. Thirteen students did not meet the inclusion criteria (they were less than 18 years or homosexual) and were excluded. In total, 345 students from HMU and 448 from TLU consented and completed the baseline interview. At this time, the CCIHP team collected the phone numbers and email addresses of these students separately from the survey so a contracted IT company could contact and invite them to log into the relevant learning program [109, 110].

#### Randomization and blinding

Using the list of students who completed the baseline survey, CCIHP staff assigned each participant a random number using the RAND function in Microsoft Excel. These random numbers were sorted in ascending order. Students in the first half of this list were randomly assigned to GlobalConsent, and students in the second half of this list were randomly assigned to the health-education attention control program. Participants and Emory personnel in the study team have been blinded to these assignments through the numeric, unlabeled coding of treatment and control allocation in the data system.

#### RealConsent program

The content of the adapted GlobalConsent will be described in a separate adaptation results paper. The original RealConsent was delivered via a secure Web portal. The program included six 30-min modules, each ranging in the number of segments [1–14] and types of activities, with diverse actors and language suitable for male students in the United States (Table 3). Each module involved didactic activities, interactivity, and episodes of a serial drama that modeled positive behaviors, such as intervening and communicating effectively with female sex partners. RealConsent was programmed so participants could work at their own pace and could not skip segments. Participants were encouraged by email to complete all modules in three weeks and were asked to give feedback on each module upon ending it to assess the extent of completion.

**Table 3:** Original RealConsent Program: Session Topics and Description of Session Content to be Adapted for Vietnam

Module	Topic	Description
1	**Consent for sex**	Serial drama episodes of young men discussing challenges of obtaining effective consent for sex; expert discussing the 4 elements of effective consent for sex; interactive segments presenting sexual scenarios related to the 4 elements of consent: young men are asked to indicate whether consent was obtained or possible, then feedback on their choices is provided; videos of real victims’ stories of being raped.
2	**Rape myths, gender roles**	Serial drama episodes of young men discussing women who they know who were raped; interactive activity of male narrator discussing 6 main rape myths and debunking each of them; video of male narrator describing the socialization process for men—“ridiculous reality” and how it contributes to sexism and VAW; female narrator describing socialization process for women and how it contributes to a culture of sexism; interactive game where several profiles of young men are provided with images and men have to choose, “who is the rapist.”
3	**Effective communi-cation**	Serial drama episodes of young men discussing challenges of communication with a young woman to have sex and how to approach; segment describing young man and woman communicating all night to show inaccurate cue perceptions, then showing correction through communication; videos of young women describing what they want in terms of communication; interactive segment w/sexual scenarios and questions around consent, alcohol, and communication.
4	**Alcohol and rape**	Serial drama episodes of young men at a house party with alcohol being served and modeling responsible behavior (e.g., deciding not to have sex); interactive segment that is a quiz with feedback given covering the physical, emotional and cognitive effects of alcohol; interactive segment of sexual scenarios involving alcohol and whether consent is possible; segment providing normative feedback on binge drinking among college students; 1st person video of young man going out for the evening and drinking alcohol and the negative outcomes.
5	**Victim empathy**	Serial drama episodes of young men discussing women’s rape experiences and men’s rape experiences in context of a rape that occurred on campus; segment highlighting inaccurate statements about rape and providing true information; video of expert discussing nuances of coercion and coercive behavior with 3 young men; real stories from a young man and young woman describing their sexual assault/rape; video clips of people providing tips on how to help a survivor.
6	**Bystander interven-tion**	Serial drama episodes of young men discussing the idea of pluralistic ignorance and doing something rather than nothing to stop a guy from being violent; video of expert discussing the barriers to intervening, safe and effective ways to intervene, and benefits to intervening; videos of 3 people who were in a situation where they did not intervene with discussion on the negative consequences; 3 videos with narration by male narrator of men behaving badly toward women and then illustrating how to intervene with positive outcomes shown.

Source [76].

#### Attention-control program

The study team developed the health-education, attention-control program, called Adolescent Health Education (AHEAD), from open-access content. Content was selected to be appropriate for young people in Vietnam. To emulate the delivery conditions of the adapted GlobalConsent, the study team developed AHEAD to be a web-based, multimedia, health promotion program with six modules. These modules included educational material on brain development, nutrition, physical activity, substance use, sleep, and agency (Table 4). Each of the six modules was designed to last 35–45 min and was audio-narrated in Vietnamese, to approximate GlobalConsent in format of delivery, intensity, and duration.

**Table 4:** Adolescent Health Education (AHEAD) Attention-Control Program: Session Topics and Description of Session Content

Module	Topic	Description of Session Content
1	**Brain development**	Explain adolescent and young adult brain maturation with tips on how to promote brain growth. Describe how brain development influences health outcomes with narration and images of developmental risks of addiction, risk taking, and peer pressure
2	**Nutrition**	Describe healthy and unhealthy eating habits, with explanations of how nutrition affects youth brain development and the negative health effects of eating disorders
3	**Physical activity**	Explain the benefits of physical activity and the recommended levels of physical activity during adolescence and adulthood. Interactive assessment of how to include physical activity in a healthy lifestyle
4	**Substance use**	Module on the prevalence of tobacco in Vietnam, different types of addictive substances, explanation on substance abuse and effects (i.e. addiction, withdrawals) and ways to prevent substance abuse
5	**Sleep**	Assess the importance of sleep and length of recommended sleep, the negative effects of sleep deprivation on different aspects of health and academics. Students explore the types and symptoms of sleep disorders, and learn strategies for better sleep habits
6	**Agency**	Explain how to develop agency when transitioning to adulthood, including the key ways to become a responsible adult and how to take responsibility for one’s medical care

#### Fidelity to intervention

GlobalConsent and AHEAD were delivered via a secure Web portal to smartphones, so human delivery errors were minimized. We were able to monitor login/logout times, frequency of logging in/out, and total time studying each module. One week after launching the learning programs, any student who had not logged in received a reminder via email and SMS to his smartphone. Students who had not completed the modules as planned (for this study, one module per week) received encouraging messages via email and smartphone. After two reminders via email and SMS, the study team at CCIHP contacted students who still had not advanced in the program by calling directly or contacting indirectly via a general Facebook group with non-named members and connections visible only to the Facebook account administrator. This behavioral data will be used to assess program completion, dose of exposure (number of modules completed for those who did not complete the program), and eventually, any refinements needed for the web application.

#### Data collection

Data on outcomes, mediators, and controls were assessed at baseline (wave 1; in September 2019) and at six months post-baseline (Wave 2; 12 weeks after finishing the last program module), and will be assessed at 12 months post-baseline (Wave 3) (Supplementary File 2. Quantitative Assessment Forms). The outcomes, exposure, mediators, and control variables are summarized in Table 5, along with the wave in which each measure has been (will be) included. We have considered confounders and modifiers, such as family background, exposure to violence in childhood, and exposure to online and other forms of explicit sexual content (Table 5). Assessment forms, scales, and questions were adapted, as needed, from the original RealConsent assessment forms and others in the field to be appropriate for the local context and to align with the adapted learning modules [66].

**Table 5:** Outcomes, Exposure, Mediators, and Control Variables

Variables	Title of Scale (source) [# items]	Example Item	Study Wave(s)
**Outcomes**			
Sexual violence (SV) perpetration	Sexual Experiences Survey [111] [48]	I watched someone while they were undressing, were nude, or were having sex, when they did not agree to it.	1–3
Bystander attitudes	Barriers to Bystander Behavior [112] [14]	If I think a woman made choices that increased her risk, I would not intervene to reduce her risk for sexual violence.	1–3
Intention to intervene	Readiness to Intervene [113] [8]	I don’t believe sexual violence is a big problem on campus.	1–3
Bystander self-efficacy	Bystander Efficacy scale [114] [11]	Express your discomfort if a guy makes a joke about a woman’s body. [Very/somewhat/not at all confident]	1–3
Bystander behavior	Bystander Intervention Behavior [112] [17]	I have asked a woman if she needed help when I noticed she was being harassed by a guy.	1–3
**Exposure**			
Treatment vs. attention control	1	Single-blinded, random assignment to theory-based, 6-session web program to prevent SV in college men (yes/no)	1
**Cognitive, Attitudinal, Affective Mediators**			
Knowledge of sexual violence	Legal Knowledge Scale [115] [22]	Taking a sexual photo or video of someone without consent [Illegal/legal but harmful/not sexual violence]	1–3
Knowledge of sexual coercion	Sexual Coercion in Intimate Relationships scale [116] [19]	Asking your dating partner repeatedly to perform a sexual act after they have said that they do not want to [is/is not coercive].	1–3
Attitudes about sexual consent	Sexual Consent scale [117] [21]	A person can express non-consent for sex at any time during sexual contact.	1–3
Restrictive attitudes about gender	Gender Equitable Men scale [118] and study team [15]	A woman should obey her husband even when she disagrees with him.	1–3
Endorsement of rape myths	Illinois Rape Myth Acceptance scale [119] & College Date Rape Attitudes and Behaviors Scale [120] [28]	In the majority of rapes, the victim is promiscuous or has a bad reputation.	1–3
Sexual communication	Developed by study team [16]	If a woman kisses me, that means she wants to have sex with me.	1–3
Rape empathy	Rape Empathy Scale [121] [15]	During a trial, I empathize more with the feelings of the rapist than of the victim	1–3
Alcohol knowledge	Developed by study team [15]	Alcohol increases the likelihood of acting aggressively toward other people [true/false]	
**Covariates**			
Demographics	[10]	Age, university, major, living situation, religion, ethnicity, relationship status, gender identity, gender expression, sexual identity	1
Child maltreatment	Child maltreatment measure validated in Vietnam [122] [27]	physical abuse (6 items), emotional abuse (7 items), sexual abuse (8 items), physical neglect (3 items), emotional neglect (4 items)	3
Online exposure to explicit sexual content	Pew Foundation’s social media survey [123] and Kids Online (128) survey module [24]	Times in prior 6 mo you have seen images, video of a woman performing a sex act in which she was choked, hit, humiliated, or forced?	2–3

#### Participant compensation

Each participant has been budgeted to receive graduated payments for completing the baseline assessment, six-month follow-up assessment, and 12-month follow-up assessment ($5 and $8 paid for Baseline and Wave 2 data collection, $10 to be paid after Wave 3 data collection; total $25) as well as $2.5 for each learning module ($15 for all six modules). University staff have been paying the students directly when they finish each survey and when they complete the learning program^1^ [124].

#### Data systems

Data systems were designed to handle the secure collection, processing, storage, and analysis of project data. Data-collection modules for each wave were built into a REDCap project and delivered at baseline to participants via tablets using the REDCap mobile app, which can collect data offline and store and transmit it securely to a server when internet connectivity is available. The web application has been transmitting data entered by each participant through an encrypted network connection to a centralized database running on a secure network and infrastructure, ensuring secure electronic data movement. Each participant was assigned a unique random number identifying him across study waves and REDCap projects. All identifiable data (names, contact info, etc.) and randomized treatment/control arm assignment has been stored with the unique ID in a separate REDCap project not accessible to the Emory study team. The Emory study team will receive randomization data only after completion of the main study analyses. CCIHP and Emory staff have created and are running applications for more refined range/consistency checks in the centralized database. Systematic data collection errors for baseline and Wave 2 data collection will be identified, resolved, and documented using the logging application in REDCap. For analyses done with external statistical software, de-identified data will be extracted and held on HIPAA compliant secure networks and computing workstations.

### Data analysis

#### Descriptive analyses

We will perform univariate analysis of all demographic, screening, mediating, outcome, and control variables for the GlobalConsent treatment group and AHEAD attention-control group, individually and combined, to explore distributions, outliers, and missingness. The associations between pairs of variables will be examined. Baseline differences in these variables between the GlobalConsent treatment group and the AHEAD attention-control condition will be tested to assess the effectiveness of randomization. Sequential exploratory and confirmatory factor analyses in random split-half samples will be performed to assess the psychometric properties of scales capturing mediators, outcomes, and control measures. Based on these analyses, final item sets will be selected to create summative scales.

#### Intent-to-treat (ITT) analysis

To assess our aims based on the randomization scheme [125], we will perform mediation analyses via path analysis [102, 126]. We will assess first whether, relative to men randomized to the AHEAD control group, men randomized to GlobalConsent will have improved mediating outcomes (H1). With reference to Fig. 3, each of these relationships is represented by path *a*, or the direct effect of treatment on the mediator*.* Testing the significance of each path allows us to assess the impact of GlobalConsent on each mediator. The standardized effect coefficients will serve as effect size measures of the treatment-to-mediator relations.

We then will assess the impact of GlobalConsent on our main outcomes (H2, H3). In Fig. 3, each of these relationships is captured by the product of the two paths, *ab*, or the indirect or mediating impact of GlobalConsent on our primary outcomes, transmitted through the individual mediators. Testing the significance of each product allows us to assess the significance of each path**.** To test these mediated effects, we will use confidence intervals derived from the bias-corrected percentile-based bootstrap approach, a resampling technique, as the valid use of regular standard errors relies on the unrealistic normality assumption, given that, the product of two normally distributed regression coefficients is, in general, not normally distributed [111, 127]. A consequence is that the power of the test is reduced [127]. The bootstrap approach is suitable here, as it allows researchers to assess the test statistics associated with these mediated effects without knowledge of their true distributions [101]. The standardized indirect effect coefficients with confidence intervals will serve as effect size measures of the various mediation effects.

**Fig. 4:**
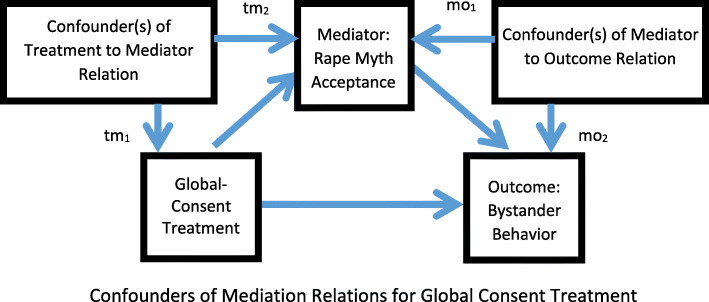
Confounders of Mediation Relations for Global Consent Treatment

We will test the robustness of the results obtained by investigating potential confounders of paths *a* and *b*, which may result in biased estimates. Figure 4 shows how confounders may affect the estimates of relationships between 1) treatment and mediator with tm_1_ and tm_2_, and 2) mediator and outcome with mo_1_ and mo_2_, which may be biased as a result of omitted or confounding variables [112, 127]. As there is randomization of the GlobalConsent treatment but not of the mediator variables, most confounders on the treatment-to-mediator relation should be eliminated. Still, we will perform a balance check on randomization using the control variables in Table 5. If imbalance on certain variables is detected, those variables may be controlled for when estimating path *a*, the GlobalConsent treatment-to-mediator relation. We also will assess the sensitivity of the results to the potential confounders on the mediator-to-outcome relation, path *b*, using a propensity score approach [113, 114]. The propensity score, or the probability that an individual receives a specified level of the mediator, given a set of observed covariates chosen from Table 5, will be estimated and used as a covariate in the mediation model to estimate path *b*. The results will be compared to those without any adjustment to detect if possible biases exist due to omitted observed confounders, and if they do, what the directions and magnitudes are. We will perform ITT analyses using the software program, Mplus [108, 115].

#### Supplemental analyses in GlobalConsent group

We will conduct several analyses in the GlobalConsent group to assist with the interpretation of findings from the ITT analyses. First, to gauge possible dose-response relationships between engagement with GlobalConsent, such as total contact time over the six-session program, and individual post-treatment mediator and outcome variables, we will examine their partial correlations for participants assigned to the GlobalConsent group, adjusting for their corresponding baseline values [114]. Second, we will assess differential program impact by examining differences in pretest-posttest change scores in mediator and outcome variables across socio-demographic characteristics, such as age, and risk groups, such online exposure to explicit sexual content.

#### Analytical issues and strategies

We note and will address relevant analytical issues. *Reports of sexual violence*. While men may systematically underreport sexual violence perpetration, our research suggests that men’s reported rate of partner-violence perpetration (37%) has been similar to women’s reported rate of partner-violence exposure (43%), with appropriate quality controls [13, 116]. Our decisions to use a tablet-assisted-self-interview platform and to store identifying information in a separate REDCap project that the analysis team cannot access should enhance disclosure by maximizing the possibility for privacy during the interview. *Differential non-participation/attrition*. We will explore the differential non-participation/attrition of men to assess the risk of response bias. With the baseline screening, socio-demographic, and other control data, we will model the response probability and use the estimated response probability to compute weights to correct for unit nonresponse using the covariates in Table 5. Estimates on the various outcomes before and after reweighting, and under different assumptions with respect to missing data, will be compared. *Item non-response*. For covariates with a non-response rate of 5% or more, we will test for differences between responders and non-responders. We may use multiple imputation, assuming the data are missing at random, to fill in missing values [117–119]. *Noncompliance***.** As some participants in the GlobalConsent group may not receive the program, we will estimate complier-average causal effect (CACE) of GlobalConsent by estimating two latent classes of compliers and non-compliers using engagement of GlobalConsent as well as covariates and control variables [120, 121]. CACE results will permit comparisons with and robustness assessments of results from the ITT analyses, and allow an examination of potential predictive factors of engagement with GlobalConsent and its intervention implications [122]. *Outliers*. We will assess the sensitivity of the results to outliers. We will exclude univariate outliers if their z scores exceed ±3. Multivariate outliers will be diagnosed using Mahalanobis distance and excluded if *p* < .0001. Results from the complete and reduced data sets will be compared. *Convergence issues and improper solutions*. We will assess the causes of any convergence issues and improper solutions when running the path analyses, such as model misspecification, non-identification, and collinearity. We will remediate by specifying suitable start values and model re-specifications [123]. *Generalizability*. To simplify logistics and to enhance feasibility of the RCT, our study sites are two universities. This strategy does not permit generalization to college men in Vietnam. Yet, the inclusion of one public university and one private university, each serving different socio-demographic groups in Vietnam, enhances the overall diversity of the sample. We will describe our sample clearly to help others understand the young men to whom study findings may be relevant and generalizable.

#### Dissemination of findings

The study team will disseminate findings through presentations at scientific meetings and peer-reviewed publications in international journals. The study team also will present key findings to university stakeholders and ministry officials in Vietnam.

## Discussion

### Summary

Sexual violence is any sexual act committed against a person without freely given consent. Among sexually experienced adolescent women 15–19 years, reports of forced sexual debut have been common globally (15%) and in the Asia/Pacific Region (14%). Early prevention among college-going men that integrates a bystander framework is a promising strategy to promote prosocial bystander behavior and to reduce sexual violence perpetration.

RealConsent is a theory-driven, evidence-based serial drama and educational program delivered in six 30-min modules via the web and tested among men attending a large, public university in the urban, Southeastern US. The current project is the first to adapt RealConsent following a systematic framework and process for adaptation of evidence-based programs. Relying on extensive formative qualitative research and feedback from local stakeholders, the study team developed the adapted program, GlobalConsent. The team is testing its impact—compared to a health-education attention control program that the study team developed for young adults in Vietnam—on sexual violence prevention and prosocial bystander behavior in college-going men in two universities in Hanoi. If GlobalConsent is effective in this middle-income setting, it has exceptional potential for national scale-up in Vietnam and for adaptation to other LMICs to prevent men’s sexual violence against women globally.

### Study limitations and strengths

Some limitations of this trial are notable. First, given the intense logistical demands of program adaptation, production, and testing, only two urban universities were included. The findings, therefore, are not generalizable to all male students at all universities in Vietnam. That said, the two study universities represent public and private institutions of higher education, diverse faculties of study, and diverse student bodies from urban and provincial areas of Vietnam. Second, a follow-up period of 12 months from baseline allows for an assessment of the short-term impact of GlobalConsent. Longer-term follow-up of study participants will be needed to assess the impacts of GlobalConsent on bystander behavior and sexually violent behavior throughout men’s time at university, and in their transition to employment and marriage. Third, similar to other web-based interventions, GlobalConsent is subject to its content becoming outdated, and updating it can require additional production costs. The team will maintain all study and program materials to mitigate these costs and the costs of all future adaptations of GlobalConsent.

Despite these limitations, if GlobalConsent is found to be effective in this sample, a large-scale implementation study of GlobalConsent in universities across Vietnam would be useful, as would studies to adapt and to test GlobalConsent in other LMICs. A major strength of GlobalConsent is that it is a web-based program, thus wide-scale implementation should be less prone to challenges with fidelity than in-person programs. Another strength of our study supporting future adaptation and implementation in other LMICs is our use of a systemic adaptation process, which allowed for the preservation of core content responsible for intervention efficacy, but flexibility to add and to tailor other elements to new populations and settings. Other major design strengths include the randomized-controlled design, a customized health-education attention-control condition, a large sample of participating university men, and refined measures to capture primary and secondary outcomes.

## Conclusion

Given high rates of sexual violence experienced by women, coupled with the growing numbers of young people – including women – attaining post-secondary education in Vietnam, colleges and universities are an ideal setting to provide sexual violence prevention programs at a critical time during adolescent development. Evidence-based sexual violence prevention programs, such as GlobalConsent, that are cost-effective, easily implemented by colleges and universities, and appealing to a diverse student population are needed in LMICs, including Vietnam. Reviews of sexual violence prevention programs describe the importance of engaging men to be women’s allies in preventing sexual violence through bystander approaches. Equally important is the ability of such programs to reach large populations rather than just male volunteers. If efficacious, GlobalConsent is a scalable intervention that, with its web-based approach, holds tremendous potential to reach large segments of male students while engaging young men to intervene now and in the future to prevent sexual violence.

## Supplementary information

**Additional file 1.** SSI and FGD Guides. Semi-Structured Interview Guides and Focus Group Discussion Guides. English language study forms for the qualitative component of the study.

**Additional file 2.** Quant Assessment Forms. Questionnaire for baseline data collection. English-language study form for the quantitative component of the study.

## Data Availability

The study protocol and study forms are being made available to the public via this open-access publication. Fully de-identified data and statistical code will be made available from the corresponding author on reasonable request (kyount@emory.edu).

## Abbreviations

AHEAD: Adolescent Health Education Program
CCIHP: Center for Creative Initiatives in Health and Population
CACE: Complier Average Causal Effect
EBIs: Evidence-based interventions
FGDs: Focus Group Discussions
HMU: Hanoi Medical University
LMICs: Low- and Middle-Income Countries
MAP: Map of the Adaptation Process
MoET: Ministry of Education and Training in Vietnam
RCT: Randomized-controlled Trial
SSI: Semi-structured Interviews
TLI: Thang Long University
WPI: whole personal impairment

## Footnotes

1 Those who did not complete all six modules were compensated according to the number of modules they completed when the learning programs were closed formally online.

## References

[1] Basile KC, Smith SG, Breiding M, Black MC, Mahendra RR. Sexual violence surveillance: uniform definitions and recommended data elements. Version 2.0. 2014.

[2] Decker MR, Latimore AD, Yasutake S, Haviland M, Ahmed S, Blum RW (2015). Gender-based violence against adolescent and young adult women in low-and middle-income countries. J Adolesc Health.

[3] Gonzales AR, Schofield RB, Schmitt GR. Sexual assault on campus: What colleges and universities are doing about it. Retrieved from National Criminal Justice Reference Service website: http://www ncjrs gov/pdffiles1/nij/205521 pdf. 2005.

[4] Amar AF, Gennaro S (2005). Dating violence in college women: associated physical injury, healthcare usage, and mental health symptoms. Nurs Res.

[5] Miller TR (1996). Victim costs and consequences: a new look: US Department of Justice, Office of Justice Programs, National Institute of ….

[6] Rennison CM (2002). Rape and sexual assault: reporting to police and medical attention, 1992–2000: US Department of Justice.

[7] Bandura A (1989). Human agency in social cognitive theory. Am Psychol.

[8] Bandura A. Social foundations of thought and action. Englewood Cliffs, NJ. 1986;1986.

[9] Hearn J (1998). The violences of men: how men talk about and how agencies respond to men's violence to women: sage.

[10] Gass JD, Stein DJ, Williams DR, Seedat S (2011). Gender differences in risk for intimate partner violence among south African adults. Journal of Interpersonal Violence.

[11] Speizer IS (2010). Intimate partner violence attitudes and experience among women and men in Uganda. Journal of interpersonal violence..

[12] Yount KM, Pham HT, Minh TH, Krause KH, Schuler SR, Anh HT (2014). Violence in childhood, attitudes about partner violence, and partner violence perpetration among men in Vietnam. Ann Epidemiol.

[13] Yount KM, Higgins EM, VanderEnde KE, Krause KH, Minh TH, Schuler SR (2016). Men’s perpetration of intimate partner violence in Vietnam: gendered social learning and the challenges of masculinity. Men Masculinities.

[14] Yount K, James-Hawkins L, Cheong Y, Naved R. Men's violence perpetration in Bangladesh: Community gender norms and violence in childhood. Psychology of Men and Masculinity Advance online publication http://dx doi org/101037/men0000069. 2016. 10.1037/men0000069 PMC5836793 29520198

[15] Rydstrøm H (2006). Masculinity and punishment: Men's upbringing of boys in rural Vietnam. Childhood..

[16] Werner J. Managing womanhoods in the family: gendered subjectivities and the state in the red river delta in Vietnam. Gender practices in contemporary Vietnam. 2004:26–46.

[17] Huou TD. Traditional families in Vietnam and the influence of Confucianism. Sociological studies on the Vietnamese family. 1991:27–55.

[18] Rydstrøm H (2003). Encountering “hot” anger: domestic violence in contemporary Vietnam. Violence against women.

[19] Rydstrøm H (2003). Embodying morality: growing up in rural northern Vietnam: University of Hawaii Press.

[20] Horton P, Rydstrom H (2011). Heterosexual masculinity in contemporary Vietnam: privileges, pleasures, and protests. Men Masculinities.

[21] Phong VH (2008). Male sexual health concerns in Muong Khen, Vietnam. Culture, health & sexuality..

[22] Rydstrøm H (2006). Sexual desires and ‘social evils’: young women in rural Vietnam. Gender, Place & Culture.

[23] Rydstrøm H, editor Sexed bodies, gendered bodies: Children and the body in Vietnam. Women's Studies International Forum; 2002: Elsevier.

[24] Ngan NT (2000). The role of men and grandparents in Vietnamese families.

[25] Cam H, Kanthoul L. ‘Teach the wife when she first arrives’ trajectories and pathways into violent and non-violent masculinities in Hue City and Phu Xuyen district. Viet Nam.

[26] Morton H, Lee HM (1996). Becoming Tongan: an ethnography of childhood: University of Hawaii Press.

[27] Werner J (2009). Gender, household and state in post-revolutionary Vietnam.

[28] Tai H-TH, Tai H-TH (2001). The country of memory: remaking the past in late socialist Vietnam: University of California Press Berkeley, CA.

[29] Phinney HM (2004). Asking for the essential child: revolutionary transformations in reproductive space in northern Viet Nam.

[30] Nguyen NN. Trends in the education sector. Economic growth, poverty, and household welfare in Vietnam. 2004:425–66.

[31] Korinek K (2004). Maternal employment during northern Vietnam's era of market reform. Social Forces.

[32] Hoang LA, Yeoh BS (2011). Breadwinning wives and “left-behind” husbands: men and masculinities in the Vietnamese transnational family. Gend Soc.

[33] Bui TC, Diamond PM, Markham C, Ross MW, Nguyen-Le T-A, Tran LHT (2010). Gender relations and sexual communication among female students in the Mekong River Delta of Vietnam. Culture, health & sexuality..

[34] Zuo X, Lou C, Gao E, Cheng Y, Niu H, Zabin LS (2012). Gender differences in adolescent premarital sexual permissiveness in three Asian cities: effects of gender-role attitudes. J Adolesc Health.

[35] Gammeltoft T, Hương NT. The politics of sexual health in Vietnam. Routledge Handbook of Sexuality Studies in East Asia: Routledge; 2014. p. 344–355.

[36] Nguyen HN, Liamputtong P (2007). Sex, love and gender norms: sexual life and experience of a group of young people in Ho Chi Minh City. Vietnam Sexual Health.

[37] Phinney HM (2008). “Rice is essential but tiresome; you should get some noodles”: Doi moi and the political economy of men’s extramarital sexual relations and marital HIV risk in Hanoi, Vietnam. Am J Public Health.

[38] Gammeltoft T (2002). Being special for somebody: urban sexualities in contemporary Vietnam. Asian Journal of Social Science.

[39] Trinh T, Steckler A, Ngo A, Ratliff E (2009). Parent communication about sexual issues with adolescents in Vietnam: content, contexts, and barriers. Sex Education.

[40] Quach T (2008). Femininity and sexual agency among young unmarried women in Hanoi. Culture, health & sexuality.

[41] Bélanger D, Hong KT (1999). Single women's experiences of sexual relationships and abortion in Hanoi. Vietnam Reproductive Health Matters.

[42] Ghuman S, Loi VM, Huy VT, Knodel J. Continuity and change in premarital sex in Vietnam. Int Fam Plan Perspect. 2006:166–74. 10.1363/3216606 17237013

[43] Do M, Fu H (2010). Attitudes toward premarital sex in contemporary Vietnam: findings from a national survey. Int J Sex Health.

[44] Jonzon R, Dang Vung N, Ringsberg KC, Krantz G (2007). Violence against women in intimate relationships: explanations and suggestions for interventions as perceived by healthcare workers, local leaders, and trusted community members in a northern district of Vietnam. Scandinavian journal of public health.

[45] Hong KT, Duong L, Nguyen N (2010). Easy to joke about, but hard to talk about: sexuality in contemporary Vietnam.

[46] Rydstrøm H (2010). Compromised ideals: family life and the recognition of women in Vietnam.

[47] Waibel G, Glück S (2013). More than 13 million: mass mobilisation and gender politics in the Vietnam Women's union. Gend Dev.

[48] Nam HLhpnV, nữ Ttnckhvp. Vietnamese women in the eighties: Foreign Languages Publishing House; 1989.

[49] Quigley J (1988). Vietnam at the legal crossroads adopts a Penal code. Am J Comp L.

[50] Huong NT (2006). Rape in Vietnam from socio-cultural and historical perspectives. Journal of Asian History.

[51] Penal CC, Ann CGS. Convention on the Elimination of All Forms of Discrimination Against Women (CEDAW)(UN Committee on the Elimination of All Forms of Discrimination Against Women), Communication No. 6/2005, UN Doc. CEDAW/C/39/D/5/2005 (1 Oct. 2007). Council of Europe Treaty Series (CETS), No. 210, 5 Nov. 2014. Marital Rape: Consent, Marriage, and Social Change in Global Context. 2016:205.

[52] Vu HS, Schuler S, Hoang TA, Quach T (2014). Divorce in the context of domestic violence against women in Vietnam. Culture, health & sexuality..

[53] James-Hawkins L, Salazar K, Hennink MM, Ha VS, Yount KM (2019). Norms of masculinity and the cultural narrative of intimate partner violence among men in Vietnam. Journal of interpersonal violence.

[54] Lonsway KA, Klaw EL, Berg DR, Waldo CR, Kothari C, Mazurek CJ (1998). Beyond “no means no” outcomes of an intensive program to train peer facilitators for campus acquaintance rape education. Journal of Interpersonal Violence..

[55] Rich MD, Utley EA, Janke K, Moldoveanu M (2010). “I'd rather be doing something else:” male resistance to rape prevention programs. The Journal of Men’s Studies.

[56] Katz J (2006). Macho paradox: why some men hurt women and and how all men can help: sourcebooks.

[57] Payne DL, Lonsway KA, Fitzgerald LF (1999). Rape myth acceptance: exploration of its structure and its measurement using theIllinois rape myth acceptance scale. J Res Pers.

[58] Amar AF, Sutherland M, Laughon K (2014). Gender differences in attitudes and beliefs associated with bystander behavior and sexual assault. Journal of forensic nursing.

[59] Newlands R, O’Donohue W (2016). A critical review of sexual violence prevention on college campuses. Acta Psychopathologica.

[60] Amar AF, Sutherland M, Kesler E (2012). Evaluation of a bystander education program. Issues in Mental Health Nursing.

[61] Amar AF, Tuccinardi N, Heislein J, Simpson S (2015). Friends helping friends: a nonrandomized control trial of a peer-based response to dating violence. Nurs Outlook.

[62] Berkowitz AD (2002). Fostering men's responsibility for preventing sexual assault.

[63] Deitz SR, Blackwell KT, Daley PC, Bentley BJ (1982). Measurement of empathy toward rape victims and rapists. J Pers Soc Psychol.

[64] Yount KM, Krause KH, Miedema SS (2017). Preventing gender-based violence victimization in adolescent girls in lower-income countries: systematic review of reviews. Soc Sci Med.

[65] DeGue S, Valle LA, Holt MK, Massetti GM, Matjasko JL, Tharp AT (2014). A systematic review of primary prevention strategies for sexual violence perpetration. Aggress Violent Behav.

[66] Tharp AT, DeGue S, Valle LA, Brookmeyer KA, Massetti GM, Matjasko JL (2013). A systematic qualitative review of risk and protective factors for sexual violence perpetration. Trauma, Violence, & Abuse..

[67] Lewis MA, Neighbors C (2007). Optimizing personalized normative feedback: the use of gender-specific referents. Journal of studies on alcohol and drugs.

[68] Vladutiu CJ, Martin SL, Macy RJ (2011). College-or university-based sexual assault prevention programs: a review of program outcomes, characteristics, and recommendations. Trauma, Violence, & Abuse.

[69] Brecklin LR, Forde DR (2001). A meta-analysis of rape education programs. Violence Vict.

[70] Gibbons R, Evans J. The evaluation of campus-based gender violence prevention programming: what we know about program effectiveness and implications for practitioners. Retrieved from National Online Resource Centre on Violence Against Women’s website: http://www vawnet org. 2013.

[71] Abraham C, Michie S (2008). A taxonomy of behavior change techniques used in interventions. Health Psychol.

[72] Salazar LF, Vivolo-Kantor A, Hardin J, Berkowitz A (2014). A web-based sexual violence bystander intervention for male college students: randomized controlled trial. J Med Internet Res.

[73] Webb T, Joseph J, Yardley L, Michie S (2010). Using the internet to promote health behavior change: a systematic review and meta-analysis of the impact of theoretical basis, use of behavior change techniques, and mode of delivery on efficacy. J Med Internet Res.

[74] Ritterband LM, Thorndike FP, Cox DJ, Kovatchev BP, Gonder-Frederick LA (2009). A behavior change model for internet interventions. Ann Behav Med.

[75] Hurling R, Fairley BW, Dias MB (2006). Internet-based exercise intervention systems: are more interactive designs better?. Psychol Health.

[76] Salazar LF, Vivolo-Kantor A, Hardin J, Berkowitz A. A web-based sexual violence bystander intervention for male college students: randomized controlled trial. Journal of medical Internet research. 2014;16(9). 10.2196/jmir.3426 PMC4180355 25198417

[77] Bennett GG, Glasgow RE (2009). The delivery of public health interventions via the internet: actualizing their potential. Annu Rev Public Health.

[78] Griffiths F, Lindenmeyer A, Powell J, Lowe P, Thorogood M (2006). Why are health care interventions delivered over the internet? A systematic review of the published literature. J Med Internet Res.

[79] Bandura A (2004). Health promotion by social cognitive means. Health Educ Behav.

[80] Fabiano PM, Perkins HW, Berkowitz A, Linkenbach J, Stark C (2003). Engaging men as social justice allies in ending violence against women: evidence for a social norms approach. J Am Coll Heal.

[81] Banyard VL, Moynihan MM, Plante EG (2007). Sexual violence prevention through bystander education: an experimental evaluation. Journal of community psychology.

[82] Ybarra ML, Thompson RE (2018). Predicting the emergence of sexual violence in adolescence. Prev Sci.

[83] Adinew YM, Hagos MA (2017). Sexual violence against female university students in Ethiopia. BMC Int Health Hum Rights.

[84] Fielding-Miller R, Shabalala F, Masuku S, Raj A. Epidemiology of campus sexual assault Among University women in Eswatini. Journal of Interpersonal Violence. 2019;0886260519888208. 10.1177/0886260519888208 PMC7231640 31738110

[85] McKleroy VS, Galbraith JS, Cummings B, Jones P, Harshbarger C, Collins C, et al. Adapting evidence–based behavioral interventions for new settings and target populations. AIDS Education & Prevention. 2006;18(supp):59–73. 10.1521/aeap.2006.18.supp.59 16987089

[86] Rogers EM (2010). Diffusion of innovations: Simon and Schuster.

[87] Salazar LF, Vivolo-Kantor A, McGroarty-Koon K (2017). Formative research with college men to inform content and messages for a web-based sexual violence prevention program. Health Commun.

[88] Gittelsohn J, Steckler A, Johnson CC, Pratt C, Grieser M, Pickrel J (2006). Formative research in school and community-based health programs and studies: “state of the art” and the TAAG approach. Health Educ Behav.

[89] Parker R, Ehrhardt AA (2001). Through an ethnographic lens: ethnographic methods, comparative analysis, and HIV/AIDS research. AIDS Behav.

[90] Needleman C, Needleman ML (1996). Qualitative methods for intervention research. Am J Ind Med.

[91] Hopson RK, Peterson JA, Lucas KJ (2001). Tales from the ‘hood’: framing HIV/AIDS prevention through intervention ethnography in the inner city. Addict Res Theory.

[92] Sivaram S, Srikrishnan AK, Latkin CA, Johnson SC, Go VF, Bentley ME (2004). Development of an opinion leader-led HIV prevention intervention among alcohol users in Chennai, India. AIDS Educ Prev.

[93] Castro FG, Barrera M, Martinez CR (2004). The cultural adaptation of prevention interventions: resolving tensions between fidelity and fit. Prev Sci.

[94] Bauman LJ, Stein RE, Ireys HT (1991). Reinventing fidelity: the transfer of social technology among settings. Am J Community Psychol.

[95] Ezzy D (1998). Lived experience and interpretation in narrative theory: experiences of living with HIV/AIDS. Qual Sociol.

[96] Hennink MM (2007). International focus group research: a handbook for the health and social sciences: Cambridge University press.

[97] Tobin GA, Begley CM (2004). Methodological rigour within a qualitative framework. J Adv Nurs.

[98] Bernard HR (2017). Research methods in anthropology: qualitative and quantitative approaches: Rowman & Littlefield.

[99] Mishler E (1986). Research interviewing: context and narrative.

[100] Glaser BG (1965). The constant comparative method of qualitative analysis. Soc Probl.

[101] Hayes AF (2017). Introduction to mediation, moderation, and conditional process analysis: a regression-based approach: Guilford publications.

[102] Baron RM, Kenny DA (1986). The moderator–mediator variable distinction in social psychological research: conceptual, strategic, and statistical considerations. J Pers Soc Psychol.

[103] Muthén LK, Muthén BO (2002). How to use a Monte Carlo study to decide on sample size and determine power. Struct Equ Model.

[104] Cohen J (1988). Statistical power analysis for the behavioral sciences: Jacob Cohen. J Am Stat Assoc.

[105] Thoemmes F, MacKinnon DP, Reiser MR (2010). Power analysis for complex mediational designs using Monte Carlo methods. Struct Equ Model.

[106] Fritz MS, MacKinnon DP (2007). Required sample size to detect the mediated effect. Psychol Sci.

[107] Wolf EJ, Harrington KM, Clark SL, Miller MW (2013). Sample size requirements for structural equation models: an evaluation of power, bias, and solution propriety. Educ Psychol Meas.

[108] Mplus ML, Guide U’s (2015). Los Angeles, CA: Muthén & Muthén; 1998.

[109] Foubert JD, Marriott KA (1997). Effects of a sexual assault peer education program on men’s belief in rape myths. Sex Roles.

[110] DiClemente R, Wingood GM (1998). Monetary incentives: a useful strategy for enhancing enrollment and promoting participation in HIV/STD risk reduction interventions.

[111] Hayes AF, Preacher KJ, Myers TA (2011). Mediation and the estimation of indirect effects in political communication research. Sourcebook for political communication research: Methods, measures, and analytical techniques.

[112] Lynch KG, Cary M, Gallop R, Ten Have TR (2008). Causal mediation analyses for randomized trials. Health Services and Outcomes Research Methodology.

[113] Rosenbaum PR, Rubin DB (1983). The central role of the propensity score in observational studies for causal effects. Biometrika..

[114] Dodds SE, Pace TW, Bell ML, Fiero M, Negi LT, Raison CL (2015). Feasibility of cognitively-based compassion training (CBCT) for breast cancer survivors: a randomized, wait list controlled pilot study. Support Care Cancer.

[115] Muthén L. Mplus user’s guide. Los Angeles: Muthén & Muthén. 1998;2015.

[116] Yount KM, Krause KH, VanderEnde KE (2016). Economic coercion and partner violence against wives in Vietnam: a unified framework?. Journal of interpersonal violence..

[117] Asparouhov T (2010). Muthén B.

[118] Enders CK (2010). Applied missing data analysis: Guilford press.

[119] Little RJ, Rubin DB (2019). Statistical analysis with missing data: John Wiley & Sons.

[120] Hewitt CE, Torgerson DJ, Miles JN. Is there another way to take account of noncompliance in randomized controlled trials? Cmaj. 2006;175(4):347-. 10.1503/cmaj.051625 PMC1534095 16908892

[121] Jo B, Asparouhov T, Muthén BO, Ialongo NS, Brown CH (2008). Cluster randomized trials with treatment noncompliance. Psychol Methods.

[122] Connell AM (2009). Employing complier average causal effect analytic methods to examine effects of randomized encouragement trials. The American journal of drug and alcohol abuse.

[123] Brown TA (2006). Confirmatory factor analysis for applied researchers.

[124] Bosnjak M, Tuten TL (2003). Prepaid and promised incentives in web surveys: an experiment. Soc Sci Comput Rev.

[125] Hollis S, Campbell F (1999). What is meant by intention to treat analysis? Survey of published randomised controlled trials. Bmj..

[126] MacKinnon D (2012). Introduction to statistical mediation analysis: Routledge.

[127] MacKinnon DP, Coxe S, Baraldi AN (2012). Guidelines for the investigation of mediating variables in business research. J Bus Psychol.

