# Supplementary material for: Preventing sexual violence in college men: a randomized-controlled trial of GlobalConsent
Source: BMC Public Health. 2020 Sep 1;20:1331. doi: 10.1186/s12889-020-09454-2 (PMC7466489; doi:10.1186/s12889-020-09454-2)
Supplement: Supplementary file 1 — Additional file 1. SSI and FGD Guides. Semi-Structured Interview Guides and Focus Group Discussion Guides. English language study forms for the qualitative component of the study. [file 12889_2020_9454_MOESM1_ESM.docx]

**GUIDE FOR FORMATIVE QUALITATIVE INTERVIEW WITH MEN**

**Participant ID #: [________________] Date: _________________________________**

**Interviewer Name: ________________________ Interview location: ______________________**

**Start time: :**

**(hour) (minutes)**

**End time: :**

**(hour) (minutes)**

**Audio file #: [___________________]**

**Notes upon completion:**

**PARTICIPANT DEMOGRAPHIC QUESTIONS**

- What province are you from?
- What is your current living situation?
  - With family/parents
  - University campus/dormitory
  - Renting flat off-campus

**OPENING QUESTIONS**

1. On a typical day, where do students like you at [university] hang out? (probe: condominiums, rent rooms for long-term living, guesthouse/hotel, private room in their parents’ house, public spaces like the cinema, coffee shops, tea shops, common rooms and spaces for students in university?)
2. What are all the words to describe different relationships that male students have with other men and women around their age? (probe: what about with young people outside the university? What about with young people inside the university?) *(Interviewer: take notes of each term until no other terms are given)*
3. According to your parents, what are the most desirable characteristics of a man? (probe: According to your male friends, what are the most desirable characteristics of a man? How do you feel about these characteristics?)
4. According to your parents, what are the most desirable characteristics of a woman? (probe: According to your male friends, what are the most desirable characteristics of a woman? How to you feel about these characteristics?)

**KEY QUESTIONS**

**Relationships between university students**

Now, I’d like to ask you more about the different relationships between men and women students at [university].

1. Can you give me a definition of [relationship term] in your own words? Can you describe a real situation with someone you know who had a [relationship term]? (probe: please think of an example from your life at university for each relationship mentioned, above; probe: how common is [term]?) (*Interviewer: If informant describes examples of terms mentioned in question 2, do not repeat here. Ask only about terms not discussed in question 2*.)
2. Now, please think about all the students at [university] you know well who are [friend-zone OR sisters-zone OR god brother/sister OR crush OR friends with benefits OR dog friends OR lover/bear]. I’d like to talk with you now about one of these kinds of relationships that you feel you know a lot about. Which type of relationship is that?
   1. Okay, what things do students do who are [relationship term selected] to show affection? (probe: kissing, hugging, sexual intercourse, etc.)
   2. What is your definition of having a sexual relationship? (probe: What is the same between sexual behaviors and behaviors showing intimacy/affection? What is different?)
   3. What sexual activities, if any, do students who are [relationship term selected] talk about?
   4. Now, tell me about a time when an uncomfortable sexual activity came up between students in a [relationship term selected] -- what happened?
   5. Now, tell me about a time when one student in a [relationship term selected] wanted to do something sexual that their partner did not what to do --- what happened? (probe: How did you learn about this event? How did it make you feel? How did you respond?)
   6. Okay, I’ve written onto cards all acts of affection or sexual activities that students do who are [relationship term selected]. Now, I would like you to put these cards into piles that make sense to you. *(Interviewer: let the informant group the cards into piles on his own; once the informant has put all of the cards into piles, ask the informant to give each of the piles a name and then to explain why each of the cards is included in a particular pile; one goal is to understand the structural taxonomy of the groupings; the other goal is to understand the social, moral, and emotional meanings that the informant attaches to each group of dating activities)*
3. In general, what should a male student at [university] expect from his [relationship term selected]? (probe: What should he expect romantically? What should he expect sexually?) (probe: In general, what should a young woman expect of her [relationship term used]? Romantically? Sexually?)

**Personal dating relationship experiences and expectations**

Next, I’d like to ask you about the relationships you have had with other women your age.

1. What kinds of relationships have you already had? (probe: which one of these would you like to talk about?)
2. Okay, I’d like to learn more about this [relationship term].
   1. How did you meet [name]? (probe: How long were you/have you been [relationship term]?)
   2. How often and where (do/did) you usually meet up with [name]?
   3. Okay, now, tell me about a time when you asked [name] to do something intimate and she agreed – what happened? (Probe: how did you know that she agreed? How did [name’s] reaction make you feel?)
   4. Now, tell me about a time when you asked [name] to do something intimate and she hesitated or disagreed – what happened? (Probe: how did you know that she hesitated or disagreed?) How did [name’s] reaction make you feel? What was your response?)
   5. Okay, now tell me about a time when [name] might have asked you to do something sexual and you hesitated or disagreed – what happened? (probe: How did [name’s] reaction make you feel?)
   6. Tell me about a time when you felt angry with [name] or when [name] felt angry with you – what exactly happened? (probe: How did it all start? *Probe for emotional, physical, or sexual forms of violence using behaviorally specific terms – yelled, called names, swore at, hit, pushed, etc*.)
   7. Okay, now tell me about a time when [name] did something that made you feel uncomfortable, sad, or hurt – what happened? (*probe for acts of following you, texting too much, calling too much, acting jealous, keeping you from seeing others, calling you names in public, etc*.)
   8. What (do/did) your male friends think about [name]? (probe: Why do you think they feel this way?)
   9. What (do/did) your parents think about [name]? (*If parents are involved, probe:* Why do you think they feel this way? What rules, if any, (do/did) your parents place on what you can or cannot do with [name]?)
   10. Tell me about a time when you broke your parents’ rules – what happened? (probe: How (are/were) your parents’ rules for [name] the same or different from your parents’ rules had for your male friendships?)

**University environment/prosocial bystander beliefs, self-efficacy, and behavior**

Now, I’d like to ask you about student gatherings at [university].

1. Was there ever a time at one of these gatherings when you saw a young man do/say something to a young woman that she did not want – What happened? (probe: How did you know it was unwanted and not agreed to? What emotions did you see from those involved? How did you feel about it? How did you respond or react?; *probe about unwanted sexual action*)
2. Was there ever a time at a student gathering when someone did something to you when you did not want to or were unable to agree or disagree – (probe: How did you know it was unwanted and not agreed to? What emotions did you see from those involved? How did you feel about it? How did you respond or react?; *probe about unwanted sexual action*)
3. Have you ever seen or experienced treatment that was unwanted in your family’s home? – What happened? (probe: How did you know it was unwanted? What emotions did you see? How did you respond or react?)

**Perceptions about rape**

Thank you for talking with me today. I have learned a lot. Now, I’d like to ask you a few more questions. Some of them may feel uncomfortable to you. Please remember that your answers are private and you may decide not to respond. Your answers are important to us and learning your views is very helpful.

1. How does a man your age know when a young woman wants to have sex? (probe: What does the man say or do? What does the woman say or do?)
2. What does the word [rape – list of terms] mean to you? (probe: *ask about each term separately; probe for each term*: how would other men your age define [rape]? How would other women your age define [rape]?)
3. As far as you know, what is the most typical situation when a rape happens between people your age? (probe: What other situations can lead to a rape? What usually is the main cause? Who most often is involved? Who typically is to blame? What are the outcomes for the perpetrator? What are the outcomes for the victim?)
4. Without saying any names, do you know of a woman your age at [university] who has had any of these kinds of experiences? (if yes, probe: Please tell me what happened.)

**CLOSING QUESTIONS**

We are nearing the end of the interview. I have a few more questions for today.

1. What are ways students your age can learn more about healthy dating and relationships at the university?
2. What do students who have had bad experiences in dating relationships do if they want to talk about it?
3. What else would you like to add that we have not yet discussed today?

Thank you for talking with me today. Your answers have been very helpful*. (Interviewer: provide comprehensive resource list to the informant. Assess emotional status of the informant and respond, as outlined in the risk assessment protocol)*.

[END RECORDING]

**GUIDE FOR FORMATIVE QUALITATIVE INTERVIEW WITH WOMEN**

**Participant ID #: [________________] Date: _________________________________**

**Interviewer Name: ________________________ Interview location: ______________________**

**Start time: :**

**(hour) (minutes)**

**End time: :**

**(hour) (minutes)**

**Audio file #: [___________________]**

**Notes upon completion:**

**PARTICIPANT DEMOGRAPHIC QUESTIONS**

- What province are you from?
- What is your current living situation?
  - With family/parents
  - University campus/dormitory
  - Renting flat off-campus

**OPENING QUESTIONS**

1. On a typical day, where do students like you at [university] hang out? (probe: condominiums, rent rooms for long-term living, guesthouse/hotel, private room in their parents’ house, public spaces like the cinema, coffee shops, tea shops, common rooms and spaces for students in university?)
2. What are all the words to describe different relationships that female students have with others? (probe: what about with young people outside the university? What about with young people inside the university?) *(Interviewer: take notes of each term until no other terms are given)*
3. According to your parents, what are the most desirable characteristics of a woman? (probe: According to your female friends, what are the most desirable characteristics of a woman? How do you feel about these characteristics?)
4. According to your parents, what are the most desirable characteristics of a man? (probe: According to your female friends, what are the most desirable characteristics of a man? How to you feel about these characteristics?)

**KEY QUESTIONS**

**Relationships between university students**

Now, I’d like to ask you more about the different relationships between men and women students at [university].

1. Can you give me a definition of [relationship term] in your own words? Can you describe a real situation with someone you know who had a [relationship term]? (probe: please think of an example from your life at university for each relationship mentioned, above; probe: how common is [term]?) (*Interviewer: If informant gives examples of terms mentioned in question 2, do not repeat here. Ask only about terms not discussed in question 2*.)
2. Now, please think about all the students at [university] you know well who are [friend-zone OR sisters-zone OR god brother/sister OR crush OR friends with benefits OR dog friends OR lover/bear]. I’d like to talk with you now about one of these kinds of relationships that you feel you know a lot about. Which type of relationship is that?
   1. Okay, what things do students do who are [relationship term selected] to show affection? (probe: kissing, hugging, sexual intercourse, etc.)
   2. What is your definition of having a sexual relationship? (probe: What is the same between sexual behaviors and behaviors showing intimacy/affection? What is different?)
   3. What sexual activities, if any, do students who are [relationship term selected] talk about?
   4. Now, tell me about a time when an uncomfortable sexual activity came up between students in a [relationship term selected] -- what happened?
   5. Now, tell me about a time when one student in a [relationship term selected] wanted to do something sexual that their partner did not what to do --- what happened? (probe: How did you learn about this event? How did it make you feel? How did you respond?)
   6. Okay, I’ve written onto cards all acts of affection or sexual activities that students do who are [relationship term selected]. Now, I would like you to put these cards into piles that make sense to you. *(Interviewer: let the informant group the cards into piles on his own; once the informant has put all of the cards into piles, ask the informant to give each of the piles a name and then to explain why each of the cards is included in a particular pile; one goal is to understand the structural taxonomy of the groupings; the other goal is to understand the social, moral, and emotional meanings that the informant attaches to each group of dating activities)*
3. In general, what should a female student at [university] expect from his [relationship term selected]? (probe: What should she expect romantically? What should she expect sexually?) (probe: In general, what should a young man expect of his [relationship term used]? Romantically? Sexually?)

**Personal dating relationship experiences and expectations**

Next, I’d like to ask you about the relationships you have had with other men your age.

1. What kinds of relationships have you already had? (probe: which one of these would you like to talk about?)
2. Okay, I’d like to learn more about this [relationship term].
   1. How did you meet [name]? (probe: How long were you/have you been [relationship term]?)
   2. How often and where (do/did) you usually meet up with [name]?
   3. Okay, now, tell me about a time when you asked [name] to do something intimate and he agreed – what happened? (Probe: how did you know that he agreed? How did [name’s] reaction make you feel?)
   4. Now, tell me about a time when you asked [name] to do something intimate and he hesitated or disagreed – what happened? (Probe: how did you know that he hesitated or disagreed?) How did [name’s] reaction make you feel? What was your response?)
   5. Okay, now tell me about a time when [name] might have asked you to do something sexual and you hesitated or disagreed – what happened? (probe: How did [name’s] reaction make you feel?)
   6. Tell me about a time when you felt angry with [name] or when [name] felt angry with you – what exactly happened? (probe: How did it all start? *Probe for emotional, physical, or sexual forms of violence using behaviorally specific terms – yelled, called names, swore at, hit, pushed, etc.*)
   7. Okay, now tell me about a time when [name] did something that made you feel uncomfortable, sad, or hurt – what happened? (*probe for acts of following you, texting too much, calling too much, acting jealous, keeping you from seeing others, calling you names in public, etc.*)
   8. What (do/did) your female friends think about [name]? (probe: Why do you think they feel this way?)
   9. What (do/did) your parents think about [name]? (*If parents are involved, probe:* Why do you think they feel this way? What rules, if any, (do/did) your parents place on what you can or cannot do with [name]?)
   10. Tell me about a time when you broke your parents’ rules – what happened? (probe: How (are/were) your parents’ rules for [name] the same or different from your parents’ rules had for your female friendships?)

**University environment/prosocial bystander beliefs, self-efficacy, and behavior**

Now, I’d like to ask you about student gatherings at [university].

1. Was there ever a time at one of these gatherings when you saw a young man do/say something to a young woman that she did not want – What happened? (probe: How did you know it was unwanted and not agreed to? What emotions did you see from those involved? How did you feel about it? How did you respond or react?; *probe about unwanted sexual action*)
2. Was there ever a time at a student gathering when someone did something to you when you did not want to or were unable to agree or disagree – (probe: How did you know it was unwanted and not agreed to? What emotions did you see from those involved? How did you feel about it? How did you respond or react?; *probe about unwanted sexual action*)

1. Have you ever seen or experienced treatment that was unwanted in your family’s home? – What happened? (probe: How did you know it was unwanted? What emotions did you see? How did you respond or react?)

**Perceptions about rape**

Thank you for talking with me today. I have learned a lot. Now, I’d like to ask you a few more questions. Some of them may feel uncomfortable to you. Please remember that your answers are private and you may decide not to respond. Your answers are important to us and learning your views is very helpful.

1. How does a woman your age show that she wants to have sex? (probe: What does the woman say or do? What does the man say or do?)
2. What does the word [rape – list of terms] mean to you? (probe: *ask about each term separately; probe for each term*: how would other men your age define [rape]? How would other women your age define [rape]?)
3. As far as you know, what is the most typical situation when a rape happens between people your age? (probe: What other situations can lead to a rape? What usually is the main cause? Who most often is involved? Who typically is to blame? What are the outcomes for the perpetrator? What are the outcomes for the victim?)
4. Without saying any names, do you know of a woman your age at [university] who has had any of these kinds of experiences? (if yes, probe: Please tell me what happened.)

**CLOSING QUESTIONS**

We are nearing the end of the interview. I have a few more questions for today.

1. What are ways students your age can learn more about healthy dating and relationships at the university?
2. What do students who have had bad experiences in dating relationships do if they want to talk about it?
3. What else would you like to add that we have not yet discussed today?

Thank you for talking with me today. Your answers have been very helpful*. (Interviewer: provide comprehensive resource list to the informant. Assess emotional status of the informant and respond, as outlined in the risk assessment protocol)*.

[END RECORDING]

**Focus Group Discussion Guide: Feedback on Original *RealConsent* Modules**

**University Men**

**Group ID #: [____________________] Date: _________________________________**

**Moderator Name: _____________________ FGD location: __________________________**

**Start time: : am/pm**

**(hour) (min)**

**End time: : am/pm**

**(hour) (min)**

**Audio file #: [___________________]**

**Modules discussed: 1-2 3-4 5-6**

**Number of participants in group: _____**

**Characteristics (up to 10 participants)**

**Participant 1: Year in school_____ Age_____ Ethnicity_______ Sexual experience (low/med/high)**

**Participant 2: Year in school_____ Age_____ Ethnicity_______ Sexual experience (low/med/high)**

**Participant 3: Year in school_____ Age_____ Ethnicity_______ Sexual experience (low/med/high)**

**Participant 4: Year in school_____ Age_____ Ethnicity_______ Sexual experience (low/med/high)**

**Participant 5: Year in school_____ Age_____ Ethnicity_______ Sexual experience (low/med/high)**

**Participant 6: Year in school_____ Age_____ Ethnicity_______ Sexual experience (low/med/high)**

**Participant 7: Year in school_____ Age_____ Ethnicity_______ Sexual experience (low/med/high)**

**Participant 8: Year in school_____ Age_____ Ethnicity_______ Sexual experience (low/med/high)**

**Participant 9: Year in school_____ Age_____ Ethnicity_______ Sexual experience (low/med/high)**

**Participant 10: Year in school_____ Age_____ Ethnicity_______ Sexual experience (low/med/high)**

**Notes upon completion:**

**__________________________________________________________________________________**

**______________________________________________________________________________________**

**______________________________________________________________________________________**

**______________________________________________________________________________________**

**______________________________________________________________________________________**

**Introductions & Warm-up**

[Facilitators greet participants and introduce themselves.]

*Thank you for agreeing to participate. I appreciate your help. Is everyone ready to begin? Great, let’s begin. Can everyone please share their first name and area of study?*

[Have all participants introduce themselves, then move into a discussion of ground rules.]

Firstly, we want everyone to feel comfortable. There are no right or wrong answers here, we just want to hear your opinions about the segments we present to you. Therefore, please be respectful of the other participants’ thoughts and opinions. We can disagree as long as we do so in a constructive manner. Secondly, we want to hear from each one of you, so if you notice that you have been contributing a lot to the discussion, take a step back and let us hear from someone who may have been speaking less. Thirdly, you may feel free to leave the room if you need to use the restroom or take a short break. Finally, your participation in this focus group discussion is voluntary, so if the content makes you feel psychologically distressed, you are free to withdraw from the study. Does anybody have any other ground rules they would like to add to ensure that this is a respectful and productive discussion?

[See if any participants have ground rules to add.]

**RealConsent Modules**

***Module 1: Consent for sex***

*Now, we are going to show you some videos for about 5-10 minutes and we would like your feedback on what you saw. Please find 1.2 and 1.3 on your Viewer’s Guide and fill in the three columns corresponding to these two sections.*

**[Participants view segments 1.2-1.3 (6.5 min) and fill in corresponding sections on Viewer’s Guide.]**

*In your opinion, what was the main message of segment 1.2 that most stood out to you? (Probe: What main message stood out for others?)*

*By a show of fingers, how relevant is this segment for young men at [your university]? (Probe: What were all of the reasons that you gave this segment the rating that you did? What was relevant/not relevant?)*

*By a show of hands, would you keep the segment as is? Remove it entirely? Change something?*

**[Invite participants who said remove or change to explain their reasoning and to offer specific changes.]**

*Now let’s move on to segment 1.3. In your opinion, what was the main message of segment 1.3 that most stood out to you? (Probe: Who has a different idea to share?)*

*By a show of fingers, how relevant is this segment for young men at [your university]? (Probe: What were all of the reasons that you gave this segment the rating that you did? What was relevant/not relevant?)*

*By a show of hands, would you keep the segment as is? Change something? Remove it entirely?*

**[Invite participants who said remove or change to explain their reasoning and to offer specific changes.]**

*Please find 1.4 and 1.5 on your Viewer’s Guide and fill in the three columns corresponding to these two sections.*

**[Participants view segments 1.4-1.5 (5.5 min) and fill in corresponding sections on Viewer’s Guide.]**

*In your opinion, what was the main message of segment 1.4 that stood out to you? (Probe: What message most stood out for others?)*

*By a show of fingers, how relevant is this segment for young men at [your university]? (Probe: What were all of the reasons that you gave this segment the rating that you did? What was relevant/not relevant?)*

*By a show of hands, would you keep the segment as is? Change something? Remove it entirely?*

**[Invite participants who said remove or change to explain their reasoning and to offer specific changes.]**

*Now let’s move on to segment 1.5. From your perspective, what was the main message of segment 1.5 that most stood out to you? (Probe: What main message most stood out for others?)*

*By a show of fingers, how relevant is this segment for young men at [your university]? (Probe: What were all of the reasons that you gave this segment the rating that you did? What was relevant/not relevant?)*

*By a show of hands, would you keep the segment as is? Change something? Remove it entirely?*

**[Invite participants who said remove or change to explain their reasoning and to offer specific changes.]**

*Please find segments 1.6 and 1.7 on your Viewer’s Guide and fill in the three columns corresponding to these two sections.*

**[Participants view segments 1.6-1.7 (7.75 min) and fill in corresponding sections on Viewer’s Guide.]**

*In your opinion, what was the main message of segment 1.6 that most stood out to you? (Probe: What most stood out for others?)*

*By a show of fingers, how relevant is this segment for young men at [your university]? (Probe: What were all of the reasons that you gave this segment the rating that you did?? What was relevant/not relevant?)*

*By a show of hands, would you keep the segment as is? Change something? Remove it entirely?*

**[Invite participants who said remove or change to explain their reasoning and to offer specific changes.]**

*Now let’s move on to segment 1.7. In your opinion, what was the main message of segment 1.7 that most stood out to you? (Probe: What other main message stood out to others?)*

*By a show of fingers, how relevant is this segment for young men at [your university]? (Probe: What were all of the reasons that you gave this segment the rating that you did?? What was relevant/not relevant?)*

*By a show of hands, would you keep the segment as is? Change something? Remove it entirely?*

**[Invite participants who said remove or change to explain their reasoning and to offer specific changes.]**

*Please find 1.8, 1.9, and 1.10 on your Viewer’s Guide and fill in the three columns corresponding to these two sections.*

**[Participants view module 1.8-1.10 (8 min) and fill in corresponding sections on Viewer’s Guide.]**

*In your opinion, what was the main message of segment 1.8 and 1.9 that most stood out to you? (Probe: What other main message stood out to others?)*

*By a show of fingers, how relevant is this segment for young men at [your university]? (Probe: What were all of the reasons that you gave this segment the rating that you did?? What was relevant/not relevant?)*

*By a show of hands, would you keep the segment as is? Change something? Remove it entirely?*

**[Invite participants who said remove or change to explain their reasoning and to offer specific changes.]**

*Now let’s move on to segment 1.10. In your opinion, what was the main message of segment 1.10 that most stood out to you? (Probe: What other main message stood out to others?)*

*By a show of fingers, how relevant is this segment for young men at [your university]? (Probe: What were all of the reasons that you gave this segment the rating that you did?? What was relevant/not relevant?)*

*By a show of hands, would you keep the segment as is? Change something? Remove it entirely?*

**[Invite participants who said remove or change to explain their reasoning and to offer specific changes.]**

**[Participants view module 1.11-1.12 (3.5 min) and fill in corresponding sections on Viewer’s Guide.]**

*In your opinion, what was the main message of segment 1.11 that most stood out to you? (Probe: What other main message stood out to others?)*

*By a show of fingers, how relevant is this segment for young men at [your university]? (Probe: What were all of the reasons that you gave this segment the rating that you did?? What was relevant/not relevant?)*

*By a show of hands, would you keep the segment as is? Change something? Remove it entirely?*

[Invite participants who said remove or change to explain their reasoning and to offer specific changes.]

*Now let’s move on to segment 1.12. In your opinion, what was the main message of segment 1.12 that most stood out to you? (Probe: What other main message stood out to others?)*

*By a show of fingers, how relevant is this segment for young men at [your university]? (Probe: What were all of the reasons that you gave this segment the rating that you did?? What was relevant/not relevant?)*

*By a show of hands, would you keep the segment as is? Change something? Remove it entirely?*

**[Invite participants who said remove or change to explain their reasoning and to offer specific changes.]**

**[End of module discussion]**

How has your definition of consent changed after viewing this module?

***Module 2: Rape myths, gender roles***

*Now we are moving on to the next module. Please turn to Module 2 on your viewer’s guide and get ready to watch segments 2.2-2.3.*

**[Participants view module 2.2-2.3 (4 min) and fill in corresponding sections on Viewer’s Guide.]**

*In your opinion, what was the main message of segment 2.2 that most stood out to you? (Probe: What other main message stood out to others?)*

*By a show of fingers, how relevant is this segment for young men at [your university]? (Probe: What were all of the reasons that you gave this segment the rating that you did?? What was relevant/not relevant?)*

*By a show of hands, would you keep the segment as is? Change something? Remove it entirely?*

**[Invite participants who said remove or change to explain their reasoning and to offer specific changes.]**

*Now let’s move on to segment 2.3. In your opinion, what was the main message of segment 2.3 that most stood out to you? (Probe: What other main message stood out to others?)*

*By a show of fingers, how relevant is this segment for young men at [your university]? (Probe: What were all of the reasons that you gave this segment the rating that you did?? What was relevant/not relevant?)*

*By a show of hands, would you keep the segment as is? Change something? Remove it entirely?*

**[Invite participants who said remove or change to explain their reasoning and to offer specific changes.]**

*Please find 2.4 on your Viewer’s Guide and fill in the three columns corresponding to these two sections.*

**[Participants view module 2.4 and fill in corresponding sections on Viewer’s Guide.]**

*In your opinion, what was the main message of segment 2.4 that most stood out to you? (Probe: What other main message stood out to others?)*

*By a show of fingers, how relevant is this segment for young men at [your university]? (Probe: What were all of the reasons that you gave this segment the rating that you did?? What was relevant/not relevant?)*

*By a show of hands, would you keep the segment as is? Change something? Remove it entirely?*

**[Invite participants who said remove or change to explain their reasoning and to offer specific changes.]**

*Please find 2.5 and 2.6 on your Viewer’s Guide and fill in the three columns corresponding to these two sections.*

**[Participants view module 2.5-2.6 (7.5 min) and fill in corresponding sections on Viewer’s Guide.]**

*In your opinion, what was the main message of segment 2.5 that most stood out to you? (Probe: What other main message stood out to others?)*

*By a show of fingers, how relevant is this segment for young men at [your university]? (Probe: What were all of the reasons that you gave this segment the rating that you did?? What was relevant/not relevant?)*

*By a show of hands, would you keep the segment as is? Change something? Remove it entirely?*

**[Invite participants who said remove or change to explain their reasoning and to offer specific changes.]**

*Now let’s move on to segment 2.6. In your opinion, what was the main message of segment 2.6 that most stood out to you? (Probe: What other main message stood out to others?)*

*By a show of fingers, how relevant is this segment for young men at [your university]? (Probe: What were all of the reasons that you gave this segment the rating that you did?? What was relevant/not relevant?)*

*By a show of hands, would you keep the segment as is? Change something? Remove it entirely?*

**[Invite participants who said remove or change to explain their reasoning and to offer specific changes.]**

*Please find 2.7 and 2.8 on your Viewer’s Guide and fill in the three columns corresponding to these two sections.*

**[Participants view module 2.7-2.8 and fill in corresponding sections on Viewer’s Guide.]**

*In your opinion, what was the main message of segment 2.7 that most stood out to you? (Probe: What other main message stood out to others?)*

*By a show of fingers, how relevant is this segment for young men at [your university]? (Probe: What were all of the reasons that you gave this segment the rating that you did?? What was relevant/not relevant?)*

*By a show of hands, would you keep the segment as is? Change something? Remove it entirely?*

**[Invite participants who said remove or change to explain their reasoning and to offer specific changes.]**

*Now let’s move on to segment 2.8. In your opinion, what was the main message of segment 2.8 that most stood out to you? (Probe: What other main message stood out to others?)*

*By a show of fingers, how relevant is this segment for young men at [your university]? (Probe: What were all of the reasons that you gave this segment the rating that you did?? What was relevant/not relevant?)*

*By a show of hands, would you keep the segment as is? Change something? Remove it entirely?*

**[Invite participants who said remove or change to explain their reasoning and to offer specific changes.]**

*Please find 2.9 on your Viewer’s Guide and fill in the three columns corresponding to these two sections.*

**[Participants view module 2.9-2.10 and fill in corresponding sections on Viewer’s Guide.]**

*In your opinion, what was the main message of segment 2.9 that most stood out to you? (Probe: What other main message stood out to others?)*

*By a show of fingers, how relevant is this segment for young men at [your university]? (Probe: What were all of the reasons that you gave this segment the rating that you did?? What was relevant/not relevant?)*

*By a show of hands, would you keep the segment as is? Change something? Remove it entirely?*

**[Invite participants who said remove or change to explain their reasoning and to offer specific changes.]**

**[End of module discussion]**

What does being a man mean to you? (Probe: How do expectations of men and women differ in the context of male/female relationships? Probe: did your opinion of masculinity change after viewing?)

How would you describe a rapist? (Probe: did your opinion change after viewing?)

**Closing**

*Thank you all for your contributions so far. I have a few more questions I would like to ask you.*

1. Is there a topic we have not yet discussed that you think should be included in the program?
2. How do you think university men would respond if a program like this were created for Vietnam?
3. What other suggestions about the program would you like to share with me?

*That was my last question. If you have any questions later, feel free to contact me. We appreciate the information you have provided and your willingness to share so openly with us and the rest of the group. Please remember the agreement you made to refrain from discussing the information shared today anywhere, or with anyone outside of this room. (“What happens in Focus Group stays in Focus Group”). At this time, we’d like to ask you to re-affirm your commitment to maintaining this confidentiality by raising your hand. Thanks again for participating in the discussion today! We appreciate you sharing your recommendations and thoughts.*

**Focus Group Discussion Guide: Feedback on Original *RealConsent* Modules**

**University Men**

**Group ID #: [____________________] Date: _________________________________**

**Moderator Name: _____________________ FGD location: __________________________**

**Start time: : am/pm**

**(hour) (min)**

**End time: : am/pm**

**(hour) (min)**

**Audio file #: [___________________]**

**Modules discussed: 1-2 3-4 5-6**

**Number of participants in group: _____**

**Characteristics (up to 10 participants)**

**Participant 1: Year in school_____ Age_____ Ethnicity_______ Sexual experience (low/med/high)**

**Participant 2: Year in school_____ Age_____ Ethnicity_______ Sexual experience (low/med/high)**

**Participant 3: Year in school_____ Age_____ Ethnicity_______ Sexual experience (low/med/high)**

**Participant 4: Year in school_____ Age_____ Ethnicity_______ Sexual experience (low/med/high)**

**Participant 5: Year in school_____ Age_____ Ethnicity_______ Sexual experience (low/med/high)**

**Participant 6: Year in school_____ Age_____ Ethnicity_______ Sexual experience (low/med/high)**

**Participant 7: Year in school_____ Age_____ Ethnicity_______ Sexual experience (low/med/high)**

**Participant 8: Year in school_____ Age_____ Ethnicity_______ Sexual experience (low/med/high)**

**Participant 9: Year in school_____ Age_____ Ethnicity_______ Sexual experience (low/med/high)**

**Participant 10: Year in school_____ Age_____ Ethnicity_______ Sexual experience (low/med/high)**

**Notes upon completion:**

**__________________________________________________________________________________**

**______________________________________________________________________________________**

**______________________________________________________________________________________**

**______________________________________________________________________________________**

**______________________________________________________________________________________**

**Introductions & Warm-up**

[Facilitators greet participants and introduce themselves.]

*Thank you for agreeing to participate. I appreciate your help. Is everyone ready to begin? Great, let’s begin. Can everyone please share their first name and area of study?*

[Have all participants introduce themselves, then move into a discussion of ground rules.]

**RealConsent Modules**

***Module 3: Effective communication***

*Now we are moving on to the next module. Please turn to Module 3 on your viewer’s guide and get ready to watch segments 3.2-3.3.*

**[Participants view module 3.2-3.3 and fill in corresponding sections on Viewer’s Guide.]**

*In your opinion, what was the main message of segment 3.2 that most stood out to you? (Probe: What other main message stood out to others?)*

*By a show of fingers, how relevant is this segment for young men at [your university]? (Probe: What were all of the reasons that you gave this segment the rating that you did?? What was relevant/not relevant?)*

*By a show of hands, would you keep the segment as is? Change something? Remove it entirely?*

**[Invite participants who said remove or change to explain their reasoning and to offer specific changes.]**

*Now let’s move on to segment 3.3. In your opinion, what was the main message of segment 3.3 that most stood out to you? (Probe: What other main message stood out to others?)*

*By a show of fingers, how relevant is this segment for young men at [your university]? (Probe: What were all of the reasons that you gave this segment the rating that you did?? What was relevant/not relevant?)*

*By a show of hands, would you keep the segment as is? Change something? Remove it entirely?*

**[Invite participants who said remove or change to explain their reasoning and to offer specific changes.]**

*Please find 3.4 and 3.5 on your Viewer’s Guide and fill in the three columns corresponding to these two sections.*

**[Participants view module 3.4-3.5 and fill in corresponding sections on Viewer’s Guide.]**

*In your opinion, what was the main message of segment 3.4 that most stood out to you? (Probe: What other main message stood out to others?)*

*By a show of fingers, how relevant is this segment for young men at [your university]? (Probe: What were all of the reasons that you gave this segment the rating that you did?? What was relevant/not relevant?)*

*By a show of hands, would you keep the segment as is? Change something? Remove it entirely?*

**[Invite participants who said remove or change to explain their reasoning and to offer specific changes.]**

*Now let’s move on to segment 3.5. In your opinion, what was the main message of segment 3.5 that most stood out to you? (Probe: What other main message stood out to others?)*

*By a show of fingers, how relevant is this segment for young men at [your university]? (Probe: What were all of the reasons that you gave this segment the rating that you did?? What was relevant/not relevant?)*

*By a show of hands, would you keep the segment as is? Change something? Remove it entirely?*

**[Invite participants who said remove or change to explain their reasoning and to offer specific changes.]**

*Please find 3.6 and 3.7 on your Viewer’s Guide and fill in the three columns corresponding to these two sections.*

**[Participants view module 3.6-3.7 and fill in corresponding sections on Viewer’s Guide.]**

*In your opinion, what was the main message of segment 3.6 that most stood out to you? (Probe: What other main message stood out to others?)*

*By a show of fingers, how relevant is this segment for young men at [your university]? (Probe: What were all of the reasons that you gave this segment the rating that you did?? What was relevant/not relevant?)*

*By a show of hands, would you keep the segment as is? Change something? Remove it entirely?*

**[Invite participants who said remove or change to explain their reasoning and to offer specific changes.]**

*Now let’s move on to segment 3.7. In your opinion, what was the main message of segment 3.7 that most stood out to you? (Probe: What other main message stood out to others?)*

*By a show of fingers, how relevant is this segment for young men at [your university]? (Probe: What were all of the reasons that you gave this segment the rating that you did?? What was relevant/not relevant?)*

*By a show of hands, would you keep the segment as is? Change something? Remove it entirely?*

**[Invite participants who said remove or change to explain their reasoning and to offer specific changes.]**

*Please find 3.8 on your Viewer’s Guide and fill in the three columns corresponding to these two sections.*

**[Participants view module 3.8-3.9 and fill in corresponding sections on Viewer’s Guide.]**

*In your opinion, what was the main message of segment 3.8 that most stood out to you? (Probe: What other main message stood out to others?)*

*By a show of fingers, how relevant is this segment for young men at [your university]? (Probe: What were all of the reasons that you gave this segment the rating that you did?? What was relevant/not relevant?)*

*By a show of hands, would you keep the segment as is? Change something? Remove it entirely?*

**[Invite participants who said remove or change to explain their reasoning and to offer specific changes.]**

**[End of module discussion]**

How similar is it to challenges you encounter in your own relationships with women, be it girlfriend/lover, casual partner, etc.? How similar/different to the way women show interest in your experience?

***Module 4: Alcohol and rape***

*Now we are moving on to the next module. Please turn to Module 4 on your viewer’s guide and get ready to watch segments 4.2-4.3.*

**[Participants view module 4.2-4.3 (3 min) and fill in corresponding sections on Viewer’s Guide.]**

*In your opinion, what was the main message of segment 4.2 that most stood out to you? (Probe: What other main message stood out to others?)*

*By a show of fingers, how relevant is this segment for young men at [your university]? (Probe: What were all of the reasons that you gave this segment the rating that you did?? What was relevant/not relevant?)*

*By a show of hands, would you keep the segment as is? Change something? Remove it entirely?*

**[Invite participants who said remove or change to explain their reasoning and to offer specific changes.]**

*Now let’s move on to segment 4.3. In your opinion, what was the main message of segment 4.3 that most stood out to you? (Probe: What other main message stood out to others?)*

*By a show of fingers, how relevant is this segment for young men at [your university]? (Probe: What were all of the reasons that you gave this segment the rating that you did?? What was relevant/not relevant?)*

*By a show of hands, would you keep the segment as is? Change something? Remove it entirely?*

**[Invite participants who said remove or change to explain their reasoning and to offer specific changes.]**

*Please find 4.4 on your Viewer’s Guide and fill in the three columns corresponding to these two sections.*

**[Participants view module 4.4 (9 min) and fill in corresponding sections on Viewer’s Guide.]**

*In your opinion, what was the main message of segment 4.4 that most stood out to you? (Probe: What other main message stood out to others?)*

*By a show of fingers, how relevant is this segment for young men at [your university]? (Probe: What were all of the reasons that you gave this segment the rating that you did?? What was relevant/not relevant?)*

*By a show of hands, would you keep the segment as is? Change something? Remove it entirely?*

**[Invite participants who said remove or change to explain their reasoning and to offer specific changes.]**

*Please find 4.5 and 4.6 on your Viewer’s Guide and fill in the three columns corresponding to these two sections.*

**[Participants view module 4.5-4.6 (2.5 min) and fill in corresponding sections on Viewer’s Guide.]**

*In your opinion, what was the main message of segment 4.5 that most stood out to you? (Probe: What other main message stood out to others?)*

*By a show of fingers, how relevant is this segment for young men at [your university]? (Probe: What were all of the reasons that you gave this segment the rating that you did?? What was relevant/not relevant?)*

*By a show of hands, would you keep the segment as is? Change something? Remove it entirely?*

**[Invite participants who said remove or change to explain their reasoning and to offer specific changes.]**

*Now let’s move on to segment 4.6. In your opinion, what was the main message of segment 4.6 that most stood out to you? (Probe: What other main message stood out to others?)*

*By a show of fingers, how relevant is this segment for young men at [your university]? (Probe: What were all of the reasons that you gave this segment the rating that you did?? What was relevant/not relevant?)*

*By a show of hands, would you keep the segment as is? Change something? Remove it entirely?*

**[Invite participants who said remove or change to explain their reasoning and to offer specific changes.]**

*Please find 4.7 and 4.8 on your Viewer’s Guide and fill in the three columns corresponding to these two sections.*

**[Participants view module 4.7-4.8 (8 min) and fill in corresponding sections on Viewer’s Guide.]**

*In your opinion, what was the main message of segment 4.7 that most stood out to you? (Probe: What other main message stood out to others?)*

*By a show of fingers, how relevant is this segment for young men at [your university]? (Probe: What were all of the reasons that you gave this segment the rating that you did?? What was relevant/not relevant?)*

*By a show of hands, would you keep the segment as is? Change something? Remove it entirely?*

**[Invite participants who said remove or change to explain their reasoning and to offer specific changes.]**

*Now let’s move on to segment 4.8. In your opinion, what was the main message of segment 4.8 that most stood out to you? (Probe: What other main message stood out to others?)*

*By a show of fingers, how relevant is this segment for young men at [your university]? (Probe: What were all of the reasons that you gave this segment the rating that you did?? What was relevant/not relevant?)*

*By a show of hands, would you keep the segment as is? Change something? Remove it entirely?*

**[Invite participants who said remove or change to explain their reasoning and to offer specific changes.]**

*Please find 4.9 on your Viewer’s Guide and fill in the three columns corresponding to these two sections.*

**[Participants view module 4.9-4.10 (3.5 min) and fill in corresponding sections on Viewer’s Guide.]**

*In your opinion, what was the main message of segment 4.9 that most stood out to you? (Probe: What other main message stood out to others?)*

*By a show of fingers, how relevant is this segment for young men at [your university]? (Probe: What were all of the reasons that you gave this segment the rating that you did?? What was relevant/not relevant?)*

*By a show of hands, would you keep the segment as is? Change something? Remove it entirely?*

**[Invite participants who said remove or change to explain their reasoning and to offer specific changes.]**

**[End of module discussion]**

In what situations, if any, does alcohol play a role in students’ lives on your campus? (Probe: making decisions, communicating with women, other substances)

**Closing**

*Thank you all for your contributions so far. I have a few more questions I would like to ask you.*

1. Is there a topic we have not yet discussed that you think should be included in the program?
2. How do you think university men would respond if a program like this were created for Vietnam?
3. What other suggestions about the program would you like to share with me?

*That was my last question. If you have any questions later, feel free to contact me. We appreciate the information you have provided and your willingness to share so openly with us and the rest of the group. Please remember the agreement you made to refrain from discussing the information shared today anywhere, or with anyone outside of this room. (“What happens in Focus Group stays in Focus Group”). At this time, we’d like to ask you to re-affirm your commitment to maintaining this confidentiality by raising your hand. Thanks again for participating in the discussion today! We appreciate you sharing your recommendations and thoughts.*

**Focus Group Discussion Guide: Feedback on Original *RealConsent* Modules**

**University Men**

**Group ID #: [____________________] Date: _________________________________**

**Moderator Name: _____________________ FGD location: __________________________**

**Start time: : am/pm**

**(hour) (min)**

**End time: : am/pm**

**(hour) (min)**

**Audio file #: [___________________]**

**Modules discussed: 1-2 3-4 5-6**

**Number of participants in group: _____**

**Characteristics (up to 10 participants)**

**Participant 1: Year in school_____ Age_____ Ethnicity_______ Sexual experience (low/med/high)**

**Participant 2: Year in school_____ Age_____ Ethnicity_______ Sexual experience (low/med/high)**

**Participant 3: Year in school_____ Age_____ Ethnicity_______ Sexual experience (low/med/high)**

**Participant 4: Year in school_____ Age_____ Ethnicity_______ Sexual experience (low/med/high)**

**Participant 5: Year in school_____ Age_____ Ethnicity_______ Sexual experience (low/med/high)**

**Participant 6: Year in school_____ Age_____ Ethnicity_______ Sexual experience (low/med/high)**

**Participant 7: Year in school_____ Age_____ Ethnicity_______ Sexual experience (low/med/high)**

**Participant 8: Year in school_____ Age_____ Ethnicity_______ Sexual experience (low/med/high)**

**Participant 9: Year in school_____ Age_____ Ethnicity_______ Sexual experience (low/med/high)**

**Participant 10: Year in school_____ Age_____ Ethnicity_______ Sexual experience (low/med/high)**

**Notes upon completion:**

**__________________________________________________________________________________**

**______________________________________________________________________________________**

**______________________________________________________________________________________**

**______________________________________________________________________________________**

**______________________________________________________________________________________**

**Introductions & Warm-up**

[Facilitators greet participants and introduce themselves.]

*Thank you for agreeing to participate. I appreciate your help. Is everyone ready to begin? Great, let’s begin. Can everyone please share their first name and area of study?*

[Have all participants introduce themselves, then move into a discussion of ground rules.]

**RealConsent Modules**

***Module 5: Victim empathy***

*Now we are moving on to the next module. Please turn to Module 5 on your viewer’s guide and get ready to watch segments 5.2-5.3.*

**[Participants view module 5.2-5.3 and fill in corresponding sections on Viewer’s Guide.]**

*In your opinion, what was the main message of segment 5.2 that most stood out to you? (Probe: What other main message stood out to others?)*

*By a show of fingers, how relevant is this segment for young men at [your university]? (Probe: What were all of the reasons that you gave this segment the rating that you did?? What was relevant/not relevant?)*

*By a show of hands, would you keep the segment as is? Change something? Remove it entirely?*

**[Invite participants who said remove or change to explain their reasoning and to offer specific changes.]**

*Now let’s move on to segment 5.3. In your opinion, what was the main message of segment 5.3 that most stood out to you? (Probe: What other main message stood out to others?)*

*By a show of fingers, how relevant is this segment for young men at [your university]? (Probe: What were all of the reasons that you gave this segment the rating that you did?? What was relevant/not relevant?)*

*By a show of hands, would you keep the segment as is? Change something? Remove it entirely?*

**[Invite participants who said remove or change to explain their reasoning and to offer specific changes.]**

*Please find 5.4 and 5.5 on your Viewer’s Guide and fill in the three columns corresponding to these two sections.*

**[Participants view module 5.4-5.5 and fill in corresponding sections on Viewer’s Guide.]**

*In your opinion, what was the main message of segment 5.4 that most stood out to you? (Probe: What other main message stood out to others?)*

*By a show of fingers, how relevant is this segment for young men at [your university]? (Probe: What were all of the reasons that you gave this segment the rating that you did?? What was relevant/not relevant?)*

*By a show of hands, would you keep the segment as is? Change something? Remove it entirely?*

**[Invite participants who said remove or change to explain their reasoning and to offer specific changes.]**

*Now let’s move on to segment 5.5. In your opinion, what was the main message of segment 5.5 that most stood out to you? (Probe: What other main message stood out to others?)*

*By a show of fingers, how relevant is this segment for young men at [your university]? (Probe: What were all of the reasons that you gave this segment the rating that you did?? What was relevant/not relevant?)*

*By a show of hands, would you keep the segment as is? Change something? Remove it entirely?*

**[Invite participants who said remove or change to explain their reasoning and to offer specific changes.]**

*Please find 5.6 on your Viewer’s Guide and fill in the three columns corresponding to these two sections.*

**[Participants view module 5.6 (6.5 min) and fill in corresponding sections on Viewer’s Guide.]**

*In your opinion, what was the main message of segment 5.6 that most stood out to you? (Probe: What other main message stood out to others?)*

*By a show of fingers, how relevant is this segment for young men at [your university]? (Probe: What were all of the reasons that you gave this segment the rating that you did?? What was relevant/not relevant?)*

*By a show of hands, would you keep the segment as is? Change something? Remove it entirely?*

**[Invite participants who said remove or change to explain their reasoning and to offer specific changes.]**

*Please find 5.7 and 5.8 on your Viewer’s Guide and fill in the three columns corresponding to these two sections.*

**[Participants view module 5.7-5.9 and fill in corresponding sections on Viewer’s Guide.]**

*In your opinion, what was the main message of segment 5.7 that most stood out to you? (Probe: What other main message stood out to others?)*

*By a show of fingers, how relevant is this segment for young men at [your university]? (Probe: What were all of the reasons that you gave this segment the rating that you did?? What was relevant/not relevant?)*

*By a show of hands, would you keep the segment as is? Change something? Remove it entirely?*

**[Invite participants who said remove or change to explain their reasoning and to offer specific changes.]**

*Now let’s move on to segment 5.8. In your opinion, what was the main message of segment 5.8 that most stood out to you? (Probe: What other main message stood out to others?)*

*By a show of fingers, how relevant is this segment for young men at [your university]? (Probe: What were all of the reasons that you gave this segment the rating that you did?? What was relevant/not relevant?)*

*By a show of hands, would you keep the segment as is? Change something? Remove it entirely?*

**[Invite participants who said remove or change to explain their reasoning and to offer specific changes.]**

**[End of module discussion]**

How would you react if someone you knew confided to you about their rape? (Probe: Did the video clip bring out any strong emotions for you?)

***Module 6: Bystander intervention***

*Now we are moving on to the next module. Please turn to Module 6 on your viewer’s guide and get ready to watch segments 6.2-6.3.*

**[Participants view module 6.2-6.3 (4.5 min) and fill in corresponding sections on Viewer’s Guide.]**

*In your opinion, what was the main message of segment 6.2 that most stood out to you? (Probe: What other main message stood out to others?)*

*By a show of fingers, how relevant is this segment for young men at [your university]? (Probe: What were all of the reasons that you gave this segment the rating that you did?? What was relevant/not relevant?)*

*By a show of hands, would you keep the segment as is? Change something? Remove it entirely?*

**[Invite participants who said remove or change to explain their reasoning and to offer specific changes.]**

*Now let’s move on to segment 6.3. In your opinion, what was the main message of segment 6.3 that most stood out to you? (Probe: What other main message stood out to others?)*

*By a show of fingers, how relevant is this segment for young men at [your university]? (Probe: What were all of the reasons that you gave this segment the rating that you did?? What was relevant/not relevant?)*

*By a show of hands, would you keep the segment as is? Change something? Remove it entirely?*

**[Invite participants who said remove or change to explain their reasoning and to offer specific changes.]**

*Please find 6.4 on your Viewer’s Guide and fill in the three columns corresponding to these two sections.*

**[Participants view module 6.4 and fill in corresponding sections on Viewer’s Guide.]**

*In your opinion, what was the main message of segment 6.4 that most stood out to you? (Probe: What other main message stood out to others?)*

*By a show of fingers, how relevant is this segment for young men at [your university]? (Probe: What were all of the reasons that you gave this segment the rating that you did?? What was relevant/not relevant?)*

*By a show of hands, would you keep the segment as is? Change something? Remove it entirely?*

**[Invite participants who said remove or change to explain their reasoning and to offer specific changes.]**

*Please find 6.5 on your Viewer’s Guide and fill in the three columns corresponding to these two sections.*

**[Participants view module 6.5 (8 min) and fill in corresponding sections on Viewer’s Guide.]**

*In your opinion, what was the main message of segment 6.5 that most stood out to you? (Probe: What other main message stood out to others?)*

*By a show of fingers, how relevant is this segment for young men at [your university]? (Probe: What were all of the reasons that you gave this segment the rating that you did?? What was relevant/not relevant?)*

*By a show of hands, would you keep the segment as is? Change something? Remove it entirely?*

**[Invite participants who said remove or change to explain their reasoning and to offer specific changes.]**

*Please find 6.6 and 6.7 on your Viewer’s Guide and fill in the three columns corresponding to these two sections.*

**[Participants view module 6.6-6.9 and fill in corresponding sections on Viewer’s Guide.]**

*In your opinion, what was the main message of segment 6.6 that most stood out to you? (Probe: What other main message stood out to others?)*

*By a show of fingers, how relevant is this segment for young men at [your university]? (Probe: What were all of the reasons that you gave this segment the rating that you did?? What was relevant/not relevant?)*

*By a show of hands, would you keep the segment as is? Change something? Remove it entirely?*

**[Invite participants who said remove or change to explain their reasoning and to offer specific changes.]**

*Now let’s move on to segment 6.7. In your opinion, what was the main message of segment 6.7 that most stood out to you? (Probe: What other main message stood out to others?)*

*By a show of fingers, how relevant is this segment for young men at [your university]? (Probe: What were all of the reasons that you gave this segment the rating that you did?? What was relevant/not relevant?)*

*By a show of hands, would you keep the segment as is? Change something? Remove it entirely?*

**[Invite participants who said remove or change to explain their reasoning and to offer specific changes.]**

*Now let’s move on to the last segment. In your opinion, what was the main message of segment 6.8 that most stood out to you? (Probe: What other main message stood out to others?)*

*By a show of fingers, how relevant is this segment for young men at [your university]? (Probe: What were all of the reasons that you gave this segment the rating that you did?? What was relevant/not relevant?)*

*By a show of hands, would you keep the segment as is? Change something? Remove it entirely?*

**[Invite participants who said remove or change to explain their reasoning and to offer specific changes.]**

**[End of module discussion]**

What are your thoughts about intervening when seeing a stranger being violent towards a woman? (Probe: What if the perpetrator was a friend? An acquaintance?) (Probe: Has your opinion changed after viewing? Probe: What would make you/men at your university more/less likely to intervene?)

**Closing**

*Thank you all for your contributions so far. I have a few more questions I would like to ask you.*

1. Is there a topic we have not yet discussed that you think should be included in the program?
2. How do you think university men would respond if a program like this were created for Vietnam?
3. What other suggestions about the program would you like to share with me?

*That was my last question. If you have any questions later, feel free to contact me. We appreciate the information you have provided and your willingness to share so openly with us and the rest of the group. Please remember the agreement you made to refrain from discussing the information shared today anywhere, or with anyone outside of this room. (“What happens in Focus Group stays in Focus Group”). At this time, we’d like to ask you to re-affirm your commitment to maintaining this confidentiality by raising your hand. Thanks again for participating in the discussion today! We appreciate you sharing your recommendations and thoughts.*

**RealConsent Participant Viewing Guide for Focus Group Discussions Modules 1 & 2**

|  | What key message or theme caught your attention in this segment? | On a scale of 1-5, how relevant is this segment to you or men at your university? (1=not at all relevant, 5=very relevant)  Comments? | Would you keep the segment as is, change something, or remove it?  If change, what needs to be changed?  If remove, why? |
| --- | --- | --- | --- |
| 1.2 WTF? Clip of guys talking about different perspectives on sexual assault and what sexual assault means to men/Duke Lacrosse jumping off point/”There’s got to be more to it than just guesswork” [2.5 mins] |  |  |  |
| 1.3 Laws and Rules in GA and at GSU, FAQs [4 min] |  |  |  |
| 1.4. It’s Not Always Who You Would Expect [0.5 min] |  |  |  |
| 1.5 Dr. Alan Berkowitz presents 4 rules of consent [5min] |  |  |  |
| 1.6 Impact of Informed Consent: Story of two guys meeting two women and the story going around campus afterward that Ben raped one of them [1 min 45 seconds] |  |  |  |
| 1.7 Informed consent scenarios: 8 scenarios followed by “is informed consent possible?” [6 min] |  |  |  |
| 1.8 Don’t be a dick: Figures acting out Dick and Jane drunkenly flirting showing their friends stepping in [<1 min] |  |  |  |
| 1.9 Show what you know: prevalence of rape on campus [0.5 min] |  |  |  |
| 1.10 Real numbers/real women [7 mins]: Female victim re: shocked, couldn’t say or do anything; female victim—thought you liked older guys; male—sister; male—size reminds woman of previous assault |  |  |  |
| 1.11 Transition segment that asks men how many rapes each year are unreported—with explanation plus statistic |  |  |  |
| 1.12 WTF-I knew someone in H.S. [2.5 min.]: Guy interrupts group of friends saying sexual assault doesn’t happen with a story of someone he knew |  |  |  |
| 1.13 Module 1 summary |  |  |  |

|  | What was the key message or theme that caught your attention in this segment? | On a scale of 1-5, how relevant is this segment to you or men at your university? (1=not at all relevant, 5=very relevant)  Comments? | Would you keep the segment as is, change something, or remove it?  If change, what needs to be changed?  If remove, why? |
| --- | --- | --- | --- |
| 2.2 WTF-Oz video clip of prison rape; “men get raped for different reasons than women get raped”; cousin raped, rape myths in action  [3 min] |  |  |  |
| 2.3 What do you think quiz: “You can tell a woman wants to hookup by the way she dresses” [1 min] |  |  |  |
| 2.4 Fact and Fiction—interactive video scenarios |  |  |  |
| 2.5 Who’s the rapist – shows that you can’t tell who a rapist is just by looking at them [2min] |  |  |  |
| 2.6 Ridiculous Reality: Peer video –Restrictive concepts of manliness, male and female gender stereotypes and what men and women get called when they step outside the box [5.5 mins] |  |  |  |
| 2.7 Priceless pep talk: Not sure what to do? What to do with a macho friend, get a new friend |  |  |  |
| 2.8 Simple Truths—short video of four women talking about their experiences with men |  |  |  |
| 2.9 WTF – Poker game; all the guys think the others are judging them for not being masculine; none of them except Sal actually want to be there [3.5 min] |  |  |  |
| 2.10 Module 2 Summary (1 min) |  |  |  |

**RealConsent Participant Viewing Guide for Focus Group Discussions Modules 3 & 4**

|  | What was the key message or theme that caught your attention in this segment? | On a scale of 1-5, how relevant is this segment to you or men at your university? (1=not at all relevant, 5=very relevant)  Comments? | Would you keep the segment as is, change something, or remove it?  If change, what needs to be changed?  If remove, why? |
| --- | --- | --- | --- |
| 3.2 WTF – all about. Discussion of date with Ellen, spent the night and made out. Discussion of different ways to make sure you have consent. [3.5min] |  |  |  |
| 3.3 Priceless pep talk – Ask; Don’t let the little head take over and assume that women want to have sex. Save yourself trouble, ask. [1 min] |  |  |  |
| 3.4 It’s not complicated—just listen |  |  |  |
| 3.5 Simple truths – women talking about deal breakers, communicating about getting tested, and no conversation [2 min] |  |  |  |
| 3.6 Yes-no-maybe: Scenario presented and question asked about whether effective consent has been met, if communication needs to happen |  |  |  |
| 3.7 Simple truths – women talking about communicating about getting tested asking about sexual history [2 min] |  |  |  |
| 3.8 WTF—good listener. Sal has a date, guys are playing video games and giving him advice about being a good listener |  |  |  |
| 3.9 Module 3 summary. Main take-away points about communication [<1min] |  |  |  |

**RealConsent Participant Viewing Guide for Focus Group Discussions Modules 5 & 6**

|  | What was the key message or theme that caught your attention in this segment? | On a scale of 1-5, how relevant is this segment to you or men at your university? (1=not at all relevant, 5=very relevant)  Comments? | Would you keep the segment as is, change something, or remove it?  If change, what needs to be changed?  If remove, why? |
| --- | --- | --- | --- |
| 4.2 WTF—Modeling Protective Strategies. Modeling drinking water, monitoring friend’s drinking, Sal and Jenny communicating  [2 min] |  |  |  |
| 4.3 Drunk driving/ doing it drunk are disastrous; sound of accident, there is no rewind button [<1 min] |  |  |  |
| 4.4 So he thinks he can drink challenge—quiz  [9 min] |  |  |  |
| 4.5 You know the consequences, step in and prevent—prosecutor  [1.25 min] |  |  |  |
| 4.6 Priceless pep talk—woman way too drunk. What should you do? Walk away  [1 min] |  |  |  |
| 4.7 Never too late—“choices” follow the path of 3 friends who went out  [7 min] |  |  |  |
| 4.8 You own your choice—“angry guy” lecture from peer; even if you’ve had a drink you make your own choices  [1 min] |  |  |  |
| 4.9 Wasted informed consent meter—quiz; 4 scenarios followed by “is this consent for sex?” [3min] |  |  |  |
| 4.10 WTF—Up early, different without a hangover; she was pretty drunk, I came back here; left his number and a note; there’s a time when people are too drunk too have sex [3.5 min] |  |  |  |
| 4.11 Module 4 Summary |  |  |  |

|  | What was the key message or theme that caught your attention in this segment? | On a scale of 1-5, how relevant is this segment to you or men at your university? (1=not at all relevant, 5=very relevant)  Comments? | Would you keep the segment as is, change something, or remove it?  If change, what needs to be changed?  If remove, why? |
| --- | --- | --- | --- |
| 5.2 WTF? Night out– The guys coming back after a night out. Talks about night with and response to her telling him about her assault. Did you hear about the guy who was arrested last night? “We’ll never know what it’s like to be raped, we’re guys”; correct those assumptions |  |  |  |
| 5.3 Rape Myths: Fact or Fiction? 4 T/F response options; “would most guys your age agree or disagree with…” [30-45 sec] |  |  |  |
| 5.4 Alan Berkowitz talking about sexual coercion and its many forms |  |  |  |
| 5.5 Transition “what do others think? |  |  |  |
| 5.6 Two Rape Stories: Can you even imagine? Woman telling a story of her assault and impacts, man telling his story of sexual assault by coach [Woman’s story is 3 min 5 sec; man’s story 3 min 15 sec (6 min 20 sec total)] (possibly Horizon) |  |  |  |
| 5.7 Aiding a Survivor |  |  |  |
| 5.8 WTF? Continuing conversation from previous WTF segment about sexual assault at a party the past night. Idiot guy (Sal) finally says he was wrong in what he said before. Mentions that that fraternity sexually assaulted their pledges last year during hazing and it was swept under the rug. [3.5min] |  |  |  |
| 5.9 Module 5 Summary |  |  |  |

|  | What was the key message or theme that caught your attention in this segment? | On a scale of 1-5, how relevant is this segment to you or men at your university? (1=not at all relevant, 5=very relevant)  Comments? | Would you keep the segment as is, change something, or remove it?  If change, what needs to be changed?  If remove, why? |
| --- | --- | --- | --- |
| 6.2 WTF? Talking about friend that won a contest to make a PSA about teenage sexual health; corrects ideas that all teenagers and college students are having sex or engage in heavy drinking, introduces concept of pluralistic ignorance [4.25 min] |  |  |  |
| 6.3 Priceless Pep Talk: Be yourself, everyone else is taken [0.25min] |  |  |  |
| 6.4 Why people don’t intervene? Three men describing situations where they didn’t intervene; Alan Berkowitz explaining why people don’t intervene; examples and explains how to intervene in each provided story (specific things to do or say) [5min 10sec] |  |  |  |
| 6.5 Stepping Up & Stepping In: Shows 3 scenarios and models bystander intervention behaviors. “You can intervene in ways that suits your personality.” [8 min] |  |  |  |
| 6.6 Rules of the Road for Intervening: guidelines for successful intervening from “Shifting Attitudes” model explained by Dr. Berkowitz [3 min] |  |  |  |
| 6.7 Transition segment: The Drunk Woman Save – Friends look out for friends, for everything else there’s Mastercard (priceless peptalk commercial) [0.5 min] |  |  |  |
| 6.8 WTF? At party mentioned in previous WTF segment, modeling Sal intervening at a party and having it go well; Sal: “I don’t want to be a sheep” [2 min 5 sec] |  |  |  |
| 6.9 Module 6 Summary |  |  |  |
