# Supplementary material for: Preventing sexual violence in college men: a randomized-controlled trial of GlobalConsent
Source: BMC Public Health. 2020 Sep 1;20:1331. doi: 10.1186/s12889-020-09454-2 (PMC7466489; doi:10.1186/s12889-020-09454-2)
Supplement: Supplementary file 2 — Additional file 2. Quant Assessment Forms. Questionnaire for baseline data collection. English-language study form for the quantitative component of the study. [file 12889_2020_9454_MOESM2_ESM.docx]

**Global Consent Baseline Survey**

*
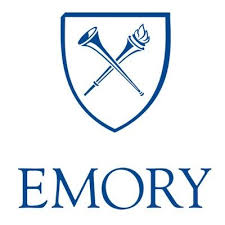

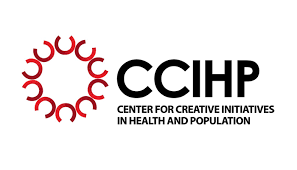
*

**Instructions:**

**Thank you for agreeing to take this survey. It should take approximately 45 minutes of your time. Please read the instructions for each section carefully.**

**Screener and Consent**

| **Question** | **Response** | **Skip** |
| --- | --- | --- |
| 1. What year are you at university? | 1, First year  0, Second year or higher | GO TO 2  GO TO 4 |
| 1. What is your age in years? | [] | <18 GO TO 4  >24 GO TO 4 |
| 1. What is your sexual orientation? | 1, Heterosexual  2, Homosexual  3, Bisexual  96, Other | GO TO 5  GO TO 5 |
| 1. Thank you for your time. At this time, you are not relevant to take part in this surey. Please return your tablet to the research assistant. |  |  |
| 1. [Insert consent form language] | 1, Yes  0, No | GO TO 4 |
| 1. Enter your ID number | [] |  |

**Demographics**

|  | **Choices** | **Skip** |
| --- | --- | --- |
| 1. Which university do you attend? | 1, Hanoi Medical University  2, Thang Long University | GO TO 3 |
| 1. What is your major? (TLU) | 1. Music 2. English Language 3. Chinies Language 4. Japanese Language 5. Korean Language 6. Business management 7. Finance and banking 8. Accounting 9. Applied Mathematics 10. Computer science 11. Communications and Computer Networks 12. Information systems 13. Nursing 14. Public Health 15. Hospital Management 16. Nutrition 17. Social work 18. Tourism and Travel Management |  |
| 1. What is your major? (HMU) | 1. Medicine 2. Traditional medicine 3. Dentomaxillofacial 4. Preventive medicine 5. Public Health 6. Medical technician 7. Nursing 8. Nutrition 9. Eye Refraction |  |
| 1. What is your ethnicity? | 0, Ethnic majority  1, Ethnic minority |  |
| 1. Please choose the option that most closely describes your current relationship status. | 1, Married  2, In a committed relationship with one or more partner(s)  3, Casually dating one or more people  4, Not currently in a sexual or romantic relationship with anyone, but have been before  5, Never been in a sexual or romantic relationship with anyone |  |
| 1. What is your religion? | 1, Buddhist  2, Christian  3, None  96, Other | GO TO 8  GO TO 8  GO TO 8 |
| 1. Please specify other religion |  |  |
| 1. Have you currently been living in Hanoi for at least one year? | 1, Yes  0, No |  |
| 1. Where do you plan to live next month in Hanoi? | 1, With parents  2, With other relatives  3, Dormitory/on campus  4, Off campus alone or with non-relatives  96, Other  98, Don't know | GO TO MODULE 1  GO TO MODULE 1  GO TO MODULE 1  GO TO MODULE 1  GO TO MODULE 1 |
| 1. Please specify other living situation |  |  |

# **Module 1.1 Knowledge about sexual violence**

| *Next is a list of sexual acts. For each sexual act, please mark whether it is illegal, legal but harmful sexual violence, or not sexual violence.* | 1,  Illegal | 2,  Legal but harmful | 3,  Not sexual violence |
| --- | --- | --- | --- |
| 1. Forcing a person to have oral sex |  |  |  |
| 1. Forcing a person to have vaginal sex |  |  |  |
| 1. Forcing a person to have anal sex |  |  |  |
| 1. A woman forcing a man to have sex |  |  |  |
| 1. Having sex with someone who is so “drunk” or “high” that they do not know the act is occurring |  |  |  |
| 1. An adult having consensual sex with a 15-year-old |  |  |  |
| 1. Forcing a spouse to have sex |  |  |  |
| 1. Forcing a person to touch your genitals |  |  |  |
| 1. Touching a person's genitals without consent |  |  |  |
| 1. An adult having sex with a 15-year-old without consent |  |  |  |
| 1. Unwanted kissing or hugging |  |  |  |
| 1. Touching a person's thigh without consent |  |  |  |
| 1. Exposing your genitals to a person without consent |  |  |  |
| 1. Sending or uploading nude photos of someone else |  |  |  |
| 1. Sending unwanted texts about sexual topics |  |  |  |
| 1. Pressuring someone to have sex |  |  |  |
| 1. Sending a picture of your genitals to someone |  |  |  |
| 1. Pressuring someone to send a nude photo of themselves |  |  |  |
| 1. Taking a sexual photo or video of someone without consent |  |  |  |
| 1. Making unwanted sexual comments about a person's body |  |  |  |
| 1. Removing someone's clothing without consent |  |  |  |
| 1. Pressuring someone to perform a sexual act with someone else |  |  |  |

## **Module 1.2 Knowledge about sexual consent**

| *Next is a set of statements about sexual consent. For each statement about sexual consent, please mark whether you totally agree, agree, are neutral, disagree, or totally disagree.* | **1,**  **Totally agree** | **2, Agree** | **3, Neutral** | **4, Disagree** | **5,**  **Totally disagree** |
| --- | --- | --- | --- | --- | --- |
| 1. Before initiating sexual intercourse, one should assume no sexual consent and verbally ask for it. |  |  |  |  |  |
| 1. Consent should be asked before ANY kind of sexual behavior, including kissing or hugging. |  |  |  |  |  |
| 1. It is just as necessary to get verbal consent for genital touching as it is for sexual intercourse. |  |  |  |  |  |
| 1. A person can express non-consent for sex at any time during sexual contact. |  |  |  |  |  |
| 1. If a person consents for sex, one can continue sexual contact even if the person changes their mind. |  |  |  |  |  |
| 1. If your partner initiates sexual contact, it is okay to continue, even if she/he is drunk. |  |  |  |  |  |
| 1. Sexual intercourse is the only sexual activity that requires explicit verbal consent. |  |  |  |  |  |
| 1. When initiating sexual contact, it is okay to assume consent and proceed until the partner says 'no' |  |  |  |  |  |
| 1. If consent for sexual intercourse has been expressed, then consent for touching sexual organs can be assumed. |  |  |  |  |  |
| 1. If a sexual request is made and the *partner* says 'no', it is okay to continue negotiating the request. |  |  |  |  |  |
| 1. The need to ask for sexual consent DECREASES as the length of a *dating relationship* INCREASES. |  |  |  |  |  |
| 1. Obtaining consent for sex is just as necessary with a casual partner as it is with a committed partner*.* |  |  |  |  |  |
| 1. If a couple has a long history of consentual sexual activity together, they no longer need to obtain consent for sex with each other. |  |  |  |  |  |
| 1. Obtaining consent for sex is just as necessary in a long-term relationship as in a new relationship. |  |  |  |  |  |
| 1. Nonverbal consent for sexual activity is okay if you've already had sex. |  |  |  |  |  |

**Module 2.1 Sexual Violence Myths**

| *Next are some statements about sexual situations between men and women. Please mark whether you totally agree, agree, are neutral, disagree, or totally disagree with each statement.* | **1,**  **Totally agree** | **2,**  **Agree** | **3, Neutral** | **4, Disagree** | **5, Totally disagree** |
| --- | --- | --- | --- | --- | --- |
| 1. Most women enjoy being submissive during sex.. |  |  |  |  |  |
| 1. If a woman dresses in a sexy dress, she is asking for sex. |  |  |  |  |  |
| 1. If a woman asks a man out on a date, then she is definitely interested in having sex. |  |  |  |  |  |
| 1. In the majority of rapes, the victim is promiscuous or has a bad reputation. |  |  |  |  |  |
| 1. A man is still entitled to have sex if his partner agreed to it but changed her mind at the last moment. |  |  |  |  |  |
| 1. Many women pretend they don't want to have sex because they don't want to appear *"easy."* |  |  |  |  |  |
| 1. A man can control his behavior no matter how sexually aroused he feels. |  |  |  |  |  |
| 1. When a woman says "no" to sex what she really means is "maybe." |  |  |  |  |  |
| 1. When a woman asks her date back to her place, she expects something sexual to happen. |  |  |  |  |  |

| *Thank you. Next are some statements about sexual violence. Please mark whether you totally agree, agree, are neutral, disagree, or totally disagree with each statement.* | **1,**  **Totally agree** | **2,**  **Agree** | **3, Neutral** | **4, Disagree** | **5, Totally disagree** |
| --- | --- | --- | --- | --- | --- |
| 1. In most cases when a woman is raped, she was asking for it. |  |  |  |  |  |
| 1. When a woman fondles a man's genitals it means she has consented to sex. |  |  |  |  |  |
| 1. If a girl is raped while she is drunk, she is at least somewhat responsible for letting things get out of hand. |  |  |  |  |  |
| 1. If a girl goes to a room alone with a guy at a party, it is her fault if she is raped. |  |  |  |  |  |
| 1. If a girl acts like a slut, eventually she is going to get into trouble. |  |  |  |  |  |
| 1. When girls get raped, it's often because they did not say "no" clearly. |  |  |  |  |  |
| 1. If a girl starts kissing a guy, she should not be surprised if he assumes she wants to have sex. |  |  |  |  |  |
| 1. When guys rape, it is usually because of their strong desire for sex. |  |  |  |  |  |
| 1. Guys don't usually intend to force sex on a girl, but sometimes they get carried away. |  |  |  |  |  |
| 1. Rape happens when a guy's sex drive gets out of control. |  |  |  |  |  |
| 1. If a girl doesn't physically resist-even if she says no-it can't be considered rape. |  |  |  |  |  |
| 1. A rape probably didn't happen if a girl doesn't have any bruises or marks.. |  |  |  |  |  |
| 1. If the accused rapist doesn't have a weapon, you really can't call it rape.. |  |  |  |  |  |
| 1. If a girl doesn't say "no" she can't claim rape. |  |  |  |  |  |
| 1. Girls who say they were raped often agreed to have sex and later regreted it. |  |  |  |  |  |

## **Module 2.2 Gender roles**

| *Next are some statements about men and women. For each statement, please mark whether you totally agree, agree, are neutral, disagree, or totally disagree.* | **1, Totally agree** | **2, Agree** | **3, Neutral** | **4, Disagree** | **5,**  **Totally disagree** |
| --- | --- | --- | --- | --- | --- |
| 1. A woman should get married before she "loses her youth". |  |  |  |  |  |
| 1. A woman's most important role is to take care of her family. |  |  |  |  |  |
| 1. A woman should obey her husband even when she disagrees with him. |  |  |  |  |  |
| 1. A woman should tolerate a violent husband to keep her family together. |  |  |  |  |  |
| 1. It is shameful for a woman to have sex before she marries. |  |  |  |  |  |
| 1. Men who help with household chores are weak. |  |  |  |  |  |
| 1. Men should be the ones to initiate dating relationships. |  |  |  |  |  |
| *Thank you. Next are some more statements about men and women. For each statement, please mark whether you totally agree, agree, are neutral, disagree, or totally disagree.* | **1, Totally agree** | **2, Agree** | **3, Neutral** | **4, Disagree** | **5,**  **Totally disagree** |
| 1. Men naturally need to have sex more often than women do. |  |  |  |  |  |
| 1. A man should have the final word on decisions in his family.. |  |  |  |  |  |
| 1. A man should be the main financial provider for his family. |  |  |  |  |  |
| 1. In young adulthood, sons should have more freedom than daughters. |  |  |  |  |  |
| 1. Men and women should share the housework, such as washing dishes, cleaning and cooking. |  |  |  |  |  |
| 1. It is more important for a woman to take care of her family than to have a career. |  |  |  |  |  |
| 1. Men and women should take equal responsibility for contraception/family planning. |  |  |  |  |  |
| 1. Men's sexual satisfaction is more important than women's sexual satisfaction. |  |  |  |  |  |

**Module 3.1. Sexual Communication**

| *The next set of statements are about sexual communication with women. For each statement, please mark whether you totally agree, agree, are neutral, disagree, or totally disagree.* | **1, Totally agree** | **2,**  **Agree** | **3, Neutral** | **4, Disagree** | **5, Totally disagree** |
| --- | --- | --- | --- | --- | --- |
| 1. Women are not supposed to talk about sex. |  |  |  |  |  |
| 1. Men should know more about sex than women. |  |  |  |  |  |
| 1. Talking about sex destroys the romance or heat of the moment. |  |  |  |  |  |
| 1. If a woman kisses me, that means she wants to have sex with me. |  |  |  |  |  |
| 1. If a woman and I have had sex before, the next time I want to have sex with her, I already have consent. |  |  |  |  |  |
| *Thank you. Next are some more statements about sexual communication with women. For each statement, please mark whether you totally agree, agree, are neutral, disagree, or totally disagree.* | **1, Totally agree** | **2,**  **Agree** | **3, Neutral** | **4, Disagree** | **5, Totally disagree** |
| 1. If a woman pushes my hand away from her, she doesn’t want me to continue touching her. |  |  |  |  |  |
| 1. If a woman refuses sex at first, she doesn’t actually mean no, and I should keep trying. |  |  |  |  |  |
| 1. Listening to a woman’s expectations about sex can help avoid assumptions in a sexual relationship. |  |  |  |  |  |
| 1. Asking a woman questions to understand her expectations about sex can help improve communication in a relationship. |  |  |  |  |  |
| 1. If I want to have sex with a woman, I need to communicate that to her. |  |  |  |  |  |

## **Module 3.2 . Skills to engage in healthy communication**

| *Next are some statements about sexual communication with dating partners. For each statement, please mark whether you are very confident, somewhat confident or not confident at all that you could do that.* | **2,**  **Very confident** | **1,**  **Somewhat Confident** | **0,**  **Not confident at all** |
| --- | --- | --- | --- |
| 1. Talking about sex with a dating partner*.* |  |  |  |
| 1. Talking about other sexual activity, such as touching, hugging, kissing, etc., with a dating partner. |  |  |  |
| 1. Disclosing personal thoughts and information, including about sex, with a dating partner. |  |  |  |
| 1. Telling my dating partner if I want to have sex. |  |  |  |
| 1. Saying no to my dating partner if I don’t want to have sex. |  |  |  |

| *Below are some questions about your dating relationships.* | | | | |
| --- | --- | --- | --- | --- |
| Have you been in a dating relationship in the past 3 months? | **1, Yes**  **2, No** | | GO TO MODULE 4 | |
| *N*ow, *please mark whether you have never, occasionally, often, or very often engaged in each of the following behaviors with any dating partner in the past three months.* | **0,**  **Never** | **1, Occasionally** | **2,**  **Often** | **3,**  **Very often** |
| 1. Talking about sex with a dating partner. |  |  |  |  |
| 1. Talking about other sexual activity, such as touching, hugging, kissing, etc., with a dating partner. |  |  |  |  |
| 1. Disclosing personal thoughts and information, including about sex, with a dating partner. |  |  |  |  |
| 1. Telling my dating partner if I want to have sex. |  |  |  |  |
| 1. Saying no to my dating partner if I don’t want to have sex. |  |  |  |  |

# **Module 4.1 How drugs and alcohol affect you**

| *Next are some statements about alcohol. Please mark whether you totally agree, agree, are neutral, disagree, or totally disagree with each statement.* | **1,**  **Totally agree** | **2,**  **Agree** | **3,**  **Neutral** | **4,**  **Disagree** | **5,**  **Totally disagree** |
| --- | --- | --- | --- | --- | --- |
| - - - 1. Alcohol impairs your ability to recall actions, including sexual activity, from the night before. |  |  |  |  |  |
| - - - 1. Alcohol increases the likelihood of acting aggressively toward other people. |  |  |  |  |  |
| - - - 1. Alcohol decreases your ability to make good decisions. |  |  |  |  |  |
| - - - 1. Alcohol affects your ability to determine a woman's intentions. |  |  |  |  |  |
| - - - 1. Alcohol affects sexual decision-making. |  |  |  |  |  |

## **Module 4.2 Alcohol and Consent**

| *Next are several statements about alcohol and consent. Please mark whether you totally agree, agree, are neutral, disagree, or totally disagree with each statement.* | **1,**  **Totally agree** | **2,**  **Agree** | **3, Neutral** | **4, Disagree** | **5, Totally disagree** | |  |
| --- | --- | --- | --- | --- | --- | --- | --- |
| 1. The more alcohol a person is drinking, the less able he/she is to consent to sexual activity. |  |  |  |  |  | |  |
| 1. A woman who is drinking alcohol heavily can still give legal consent to sexual activity. |  |  |  |  |  | |  |
| 1. If a person who has been drinking alcohol becomes sleepy or unconscious, he/she cannot give consent to any sexual activity. |  |  |  |  |  | |  |
| 1. Verbal communication is easily misunderstood when alcohol is involved in sexual activity. |  |  |  |  |  | |  |
| 1. Physical communication is easily mis-understood when alcohol is involved in sexual activity. |  |  |  |  |  | |  |
| *Thank you. Next are several statements about alcohol and sex. For each statement, please mark whether you totally agree, agree, are neutral, disagree, or totally disagree.* | **1, Totally agree** | **2,**  **Agree** | **3,**  **Neutral** | **4, Disagree** | | **5, Totally disagree** | |
| 1. Consensual drunk sex is a normal and harmless part of university life. |  |  |  |  | |  | |
| 1. When a person is drinking alcohol, he or she is implying interest in engaging in sexual activity. |  |  |  |  | |  | |
| 1. It shouldn't be considered rape if a guy is drunk and didn't realize what he was doing. |  |  |  |  | |  | |
| 1. If both partners are drunk and have sex, there is no way the man can be accused of rape or other forms of sexual violence. |  |  |  |  | |  | |
| 1. A person who is sexually assaulted after drinking alcohol should only blame him or herself. |  |  |  |  | |  | |

# **Module 5.1 Rape empathy**

| *Each of the following questions presents two statements. For each question, please mark the statement with which you most agree. There is no right or wrong answer. Please simply choose the answer with which you most agree.* |
| --- |
| 1. 1, Raping a woman is never justified.   2, Raping a woman is sometimes justified. |
| 1. 1, In a rape case, the sexual history of the reported victim is more important than that of the reported rapist.   2, In a rape case, the sexual history of the reported rapist is more important than that of the reported rape victim. |
| 1. 1, The victim of a rape usually provokes it.   2, The victim of a rape never provokes it. |
| 1. 1, I understand better how a rapist might feel than how a victim might feel during a rape.   2, I understand better how a victim might feel than how a rapist might feel during a rape. |
| 1. 1, I can understand why a man sometimes uses force to have sex with a woman.   2, I cannot understand why a man ever uses force to have sex with a woman. |
| 1. 1, When a woman dresses in a sexually attractive way, she must accept rape as a consequence.   2, When a woman dresses in a sexually attractive way, she should never have to tolerate rape. |
| 1. 1, During a trial, I empathize more with the feelings of the rapist than of the victim.   2, During a trial, I empathize more with the feelings of the victim than of the rapist. |
| 1. 1, A man is justified to rape a woman who has sex with other men.   2, A man is not justified to rape a woman who has sex with other men. |
| *Next some more questions with two statements. For each question, please choose the statement number with which you most agree. There is no right or wrong answer. Please simply choose the answer with which you most agree.* |
| 1. 1, It is up to the victim to prove that she was raped.   2, It is up to the rapist to prove he is not guilty. |
| 1. 1, It is impossible for a rape victim to enjoy being raped.   2, It is possible for a rape victim to enjoy being raped. |
| 1. 1, I understand the helplessness a rapist might feel during a rape, since he cannot control his actions.   2, I understand the helplessness a victim might feel during a rape. |
| 1. 1, A rape victim suffers more emotional trauma in dealing with the police than a rapist.   2, A rapist suffers more emotional trauma in dealing with the police than the rape victim. |
| 1. 1, It is impossible for a man to rape a woman unless she is willing.   2, It is possible for a man to rape a woman against her will. |
| 1. 1, If a rape trial were publicized, the rape victim would suffer more emotional trauma than the rapist.   2, If a rape trial were publicized, the rapist would suffer more emotional trauma than the rape victim. |
| 1. 1, If a couple has had sex, it is no longer possible for the man to rape the woman.   2, If a couple has had sex, it is rape if the man forces the woman to have sex. |
| 1. 1, A husband does not have the right to force his wife to have sex with him.   2, A husband has every right to force his wife to have sex with him. |
| 1. 1, I believe women who accuse men of rape since it takes courage to make that accusation.   2, I doubt women who accuse men of rape, since the blame is usually on both sides. |

## **Module 5.3 Sexual Coercion**

| *Thank you. Next are a list of sexual behaviors. Please mark yes, no, or unsure to whether the following behaviors are sexually coercive in a dating relationship.* | **1,**  **Yes** | **0,**  **No** | **2, Unsure** |
| --- | --- | --- | --- |
| 1. Saying that you will break up with your dating partner if they do not have sex. |  |  |  |
| 1. Threatening your dating partner with physical harm if they do not have sex. |  |  |  |
| 1. Asking your dating partner to perform a sexual act after they have said that they do not want to. |  |  |  |
| 1. Asking your dating partner repeatedly to perform a sexual act after they have said that they do not want to. |  |  |  |
| 1. Saying that you will withhold help from your dating partner if they do not have sex. |  |  |  |
| 1. Withholding help from your dating partner if they do not have sex. |  |  |  |
| 1. Promising to give your dating partner a gift in exchange for sex. |  |  |  |
| 1. Reminding your dating partner of gifts you have given them so that they feel obliged to have sex. |  |  |  |
| 1. Saying that you will have sex with someone else if your dating partner does not have sex with you. |  |  |  |
| 1. Saying that you will start a relationship with someone else if your dating partner does not have sex with you. |  |  |  |
| *Please mark yes, no, or unsure to whether the following behaviors are sexually coercive in a dating relationship.* |  |  |  |
| 1. Saying that a person would have sex with you if they were truly committed to the dating relationship. |  |  |  |
| 1. Saying that a person would have sex with you if they truly loved you. |  |  |  |
| 1. Saying you will physically harm yourself if a person does not have sex with you. |  |  |  |
| 1. Saying you will physically harm someone else if a person does not have sex with you. |  |  |  |
| 1. Saying that other people are having sex to convince someone to have sex with you. |  |  |  |
| 1. Telling a dating partner that it is their obligation to have sex with you. |  |  |  |
| 1. Being disappointed because a dating partner will not have sex with you. |  |  |  |
| 1. Asking a casual or dating partner if they are ready to have sex. |  |  |  |
| 1. Being honest with a dating partner about your sexual desires. |  |  |  |

# **Module 6.1 Responses to situations**

| *Next are some statements about intervening to prevent sexual violence. Please mark whether you totally agree, agree, are neutral to, disagree, or totally disagree with each statement.* | **1,**  **Totally agree** | **2,**  **Agree** | **3, Neutral** | **4, Disagree** | **5, Totally disagree** |
| --- | --- | --- | --- | --- | --- |
| 1. Even if I thought a woman was at risk for sexual violence, I would let others intervene. |  |  |  |  |  |
| 1. If I saw a stranger at risk for sexual violence, I would let her friends intervene. |  |  |  |  |  |
| 1. If I think a woman made choices that increased her risk, I would not intervene to reduce her risk for sexual violence. |  |  |  |  |  |
| 1. If a woman dresses or acts *provocatively*, I would not intervene to prevent others from taking *sexual advantage* of her. |  |  |  |  |  |
| 1. If a woman is drunk, I would not intervene to prevent others from taking *sexual advantage* of her. |  |  |  |  |  |
| 1. Although I would like to intervene when a guy’s sexual behavior is disturbing, I would not know what to do. |  |  |  |  |  |
| 1. Even if I thought it was y *responsibility* to help to prevent sexual violence, I would not know how to help. |  |  |  |  |  |
| 1. Even if I thought a situation might be high in sexual violence risk, I probably wouldn’t say or do anything if other people appeared unconcerned. |  |  |  |  |  |

| *Thank you. Next some more statements about intervening to prevent sexual violence. Please mark whether you totally agree, agree, are neutral to, disagree, or totally disagree with each statement.* | **1, Totally agree** | **2, Agree** | **3, Neutral** | **4, Disagree** | **5, Totally disagree** |
| --- | --- | --- | --- | --- | --- |
| 1. I hesitate to intervene when a guy’s sexual behavior is disturbing because people may not support me. |  |  |  |  |  |
| 1. Even if I thought it was my duty to intervene to prevent sexual violence, I might not to avoid looking foolish. |  |  |  |  |  |
| 1. Helping to prevent sexual violence could make people mad at me. |  |  |  |  |  |
| 1. I might get in trouble if I help to prevent sexual violence. |  |  |  |  |  |
| 1. People will think I want to start drama if I help to prevent sexual violence. |  |  |  |  |  |
| 1. I might get made fun of or picked on if I help to prevent sexual violence. |  |  |  |  |  |

## **Module 6.2 Readiness to intervene**

| *Next are some actions about intervening to prevent sexual violence. Please mark if you are very confident, somewhat confident, or not at all confident in your ability to do the following things.* | **2,**  **Very confident** | **1, Somewhat Confident** | **0,**  **Not confident at all** |
| --- | --- | --- | --- |
| 1. Express your discomfort if a guy makes a joke about a woman's body. |  |  |  |
| 1. Express your discomfort if a guy says that rape victims are to blame for being raped. |  |  |  |
| 1. Call for help if you hear a woman on campus yelling "help". |  |  |  |
| 1. Offer your help to a woman in a violent dating relationship. |  |  |  |
| *Next are some more actions about intervening to prevent sexual violence. Please mark if you are very confident, somewhat confident, or not at all confident in your ability to do the following things.* | **2,**  **Very confident** | **1, Somewhat Confident** | **0,**  **Not confident at all** |
| 1. *Report (to an authority/formally)* a guy who tells you that they had sex with someone who was passed out. |  |  |  |
| 1. *Report (to an authority/formally)* a guy who tells you that they had sex with someone who didn't give consent. |  |  |  |
| 1. *Report (to an authority/formally)* a group of drunk men at a party who are taking a drunk girl to a bedroom. |  |  |  |
| 1. Talk with authorities about information you have that might help in a sexual violence case, even if pressured by your peers to stay silent. |  |  |  |
| 1. Speak up to a guy who is using disrespectful language to describe their girlfriend. |  |  |  |
| 1. Speak up to a guy who is making excuses for using physical force in a sexual relationship. |  |  |  |
| 1. Speak up to a guy who is making excuses for forcing someone to have sex with him. |  |  |  |

| *Thank you. Now, for the next set of statements, please tell me if you find each statement about you to be true, untrue or unsure.* | **1,**  **True** | **2, Unsure** | **0,**  **Untrue** |
| --- | --- | --- | --- |
| 1. I don’t believe sexual violence is a big problem on campus. |  |  |  |
| 1. I don’t believe there is much I can do about sexual violence on campus. |  |  |  |
| 1. There isn’t much need for me to think about sexual violence on campus. |  |  |  |
| 1. Sometimes I think I should learn more about sexual violence on campus. . |  |  |  |
| 1. I am planning to learn more about the problem of sexual violence on campus. |  |  |  |
| 1. I am planning to find out what I can do about sexual violence on campus. |  |  |  |
| 1. I have recently taken part in activities or volunteered my time on projects focused on ending sexual violence on campus. |  |  |  |
| 1. I have been or am now involved in efforts to end sexual violence on campus. |  |  |  |

## **Module 6.3 Intervention strategies**

| *Below are behaviors around intervening to prevent sexual violence. Please mark if you have done each behavior more than once, once, or if you have never done that behavior in the last 12 MONTHS. If you have not been in a situation like that in the last 12 months, please select 'No opportunity'.* | **2,**  **Yes, more than once** | **1,**  **Yes, once** | **0,**  **No, I have never done this** | **9,**  **No opportunity** |
| --- | --- | --- | --- | --- |
| 1. I have encouraged others to learn more and get involved in preventing sexual or dating violence. |  |  |  |  |
| 1. I have talked with someone about sexual or dating violence as an issue for our university. |  |  |  |  |
| 1. I have talked with someone about sexually violent behaviors and warning signs. |  |  |  |  |
| 1. I have told guys not to talk about women in *sexually degrading* ways. |  |  |  |  |
| 1. I have asked a woman if she needed help when I noticed she was being harassed by a guy. |  |  |  |  |
| 1. I have told a guy who is bombarding a woman with texts that his behavior is inappropriate. |  |  |  |  |
| 1. I have told a guy who keeps asking a woman out with him after she's said no several times that his behavior is inappropriate. |  |  |  |  |
| 1. I have told a guy who is asking a girl repeatedly for sex that his behavior is sexual violence. |  |  |  |  |

| *Thank you. Next is a list of behaviors around intervening to prevent sexual violence. Please tell me if you have done each behavior more than once, once, or if you have never in the last 12 MONTHS. If you have not been in a situation like that in the last 12 months, please select 'No opportunity'.* | **2,**  **Yes, more than once** | **1,**  **Yes, once** | **0,**  **No, I have never done this** | **9,**  **No opportunity** |
| --- | --- | --- | --- | --- |
| 1. I have expressed concern or offered help to a woman who said she had an unwanted sexual experience but didn't call it 'rape'. |  |  |  |  |
| 1. I have let a woman I thought was in a violent dating relationship know I was there to help. |  |  |  |  |
| 1. I have let a woman I suspected had been sexually assaulted know I was there to help |  |  |  |  |
| 1. I have reported formally to an authority a guy who used physical force against a woman. |  |  |  |  |
| 1. I have reported formally to an authority a guy who was talking about getting a woman drunk in order to have sex with her. |  |  |  |  |
| 1. I have stopped guys from doing things that meet the definition of sexual violence. |  |  |  |  |
| 1. I have intervened with a guy’s action because I wanted to stop an act of sexual violence. |  |  |  |  |
| 1. I have intervened when I have seen a guy trying to have sex with a woman who was drunk. |  |  |  |  |
| 1. I have asked others for help in intervening to stop a guy trying to have sex with a woman who was drunk. |  |  |  |  |

**Module 7. Sexual Experiences**

| The following questions concern sexual experiences. These questions are personal, so your information is kept private. We hope this helps you to feel comfortable answering each question honestly. Select the option showing the number of times each experience has happened **in the last 6 months**. | **1, 0 times** | **2, 1 time** | | **3, 2 times** | | **4, 3 or more times** |
| --- | --- | --- | --- | --- | --- | --- |
| 1. I stared at someone in a sexual way or looked at the sexual parts of their body after they had asked me to stop. |  |  | |  | |  |
| 1. I made teasing comments of a sexual nature about someone’s body or appearance after they had asked me to stop. |  |  | |  | |  |
| 1. I sent someone sexual or obscene materials such as pictures, jokes, or stories in the mail or over the Internet, after they had asked me to stop. |  |  | |  | |  |
| 1. I showed someone pornographic pictures when they had not agreed to look at them. |  |  | |  | |  |
| 1. I made sexual or obscene phone calls to someone when they had not agreed to talk with me. |  |  | |  | |  |
| 1. I watched someone while they were undressing, were nude, or were having sex, when they did not agree to it. |  |  | |  | |  |
| 1. I took photos or videotapes of someone while they were undressing, were nude, or were having sex, when they did not agree to it. |  |  | |  | |  |
| 1. I showed someone the private areas of my body (ex. butt, penis) when they did not agree to it. |  |  | |  | |  |
| 1. I made sexual motions to someone, *such as* grabbing my crotch, pretending to masturbate, or imitating oral sex when they did not agree to it. |  |  | |  | |  |
| 1. I masturbated in front of someone when they did not agree to it. |  |  | |  | |  |
| Thank you. Next are 7 sexual experiences, each followed by 5 statements explaining how the experience may have happened. Please mark how often each experience happened to you by each means **in the past 6 months**. We are interested in the frequency of each means, whether or not they happened on the same sexual experience. Let’s begin | **1, 0 times** | **2, 1 time** | | **3, 2 times** | | **4, 3 or more times** |
| 1. **I fondled, kissed, or rubbed up against the private areas of someone’s body (lips, breast/chest, crotch or butt) or removed some of their clothes when they did not agree to it *(but did not attempt sexual penetration)* by:** |  | | | | | |
| 1. Telling lies, threatening to end the relationship, threatening to spread rumors about them, making false promises, or verbally pressuring them after they said they didn’t want to. |  |  | |  | |  |
| 1. Showing displeasure, criticizing their sexuality or attractiveness, getting angry but not using physical force after they said they didn’t want to*.* |  |  | |  | |  |
| 1. Taking advantage when they were too drunk or out of it to stop what was happening. |  |  | |  | |  |
| 1. Threatening physical harm to them or someone close to them. |  |  | |  | |  |
| 1. Using force, like holding them down with my body weight, pinning their arms, or having a weapon. |  |  | |  | |  |
| 1. **I had oral sex with someone or had someone perform oral sex on me when they did not agree to it by:** |  | | | | | |
| 1. Telling lies, threatening to end the relationship, threatening to spread rumors about them, making false promises, or verbally pressuring them after they said they didn’t want to. |  | |  | |  |  |
| 1. Showing displeasure, criticizing their sexuality or attractiveness, getting angry but not using physical force after they said they didn’t want to*.* |  | |  | |  |  |
| 1. Taking advantage when they were too drunk or out of it to stop what was happening. |  | |  | |  |  |
| 1. Threatening physical harm to them or someone close to them. |  | |  | |  |  |
| 1. Using force, like holding them down with my body weight, pinning their arms, or having a weapon. |  | |  | |  |  |
| 1. **I put my penis, fingers or objects into a woman’s vagina when she did not agree to it by:** |  | | | | | |
| 1. Telling lies, threatening to end the relationship, threatening to spread rumors about them, making false promises, or verbally pressuring them after they said they didn’t want to. |  | |  | |  |  |
| 1. Showing displeasure, criticizing their sexuality or attractiveness, getting angry but not using physical force after they said they didn’t want to |  | |  | |  |  |
| 1. Taking advantage when they were too drunk or out of it to stop what was happening. |  | |  | |  |  |
| 1. Threatening physical harm to them or someone close to them. |  | |  | |  |  |
| 1. Using force, like holding them down with my body weight, pinning their arms, or having a weapon. |  | |  | |  |  |
| 1. **I put in my penis, fingers or objects into someone’s butt when they did not agree to it by:** |  | | | | | |
| 1. Telling lies, threatening to end the relationship, threatening to spread rumors about them, making false promises, or verbally pressuring them after they said they didn’t want to. |  | |  | |  |  |
| 1. Showing displeasure, criticizing their sexuality or attractive-ness, getting angry but not using physical force after they said they didn’t want to*.* |  | |  | |  |  |
| 1. Taking advantage when they were too drunk or out of it to stop what was happening. |  | |  | |  |  |
| 1. Threatening physical harm to them or someone close to them. |  | |  | |  |  |
| 1. Using force, like holding them down with my body weight, pinning their arms, or having a weapon. |  | |  | |  |  |
| 1. **I ATTEMPTED (but did not) have oral sex with someone or make them have oral sex with me when they did not agree to it by:** |  | | | | | |
| 1. Telling lies, threatening to end the relationship, threatening to spread rumors about them, making false promises, or verbally pressuring them after they said they didn’t want to. |  | |  | |  |  |
| 1. Showing displeasure, criticizing their sexuality or attractiveness, getting angry but not using physical force after they said they didn’t want to. |  | |  | |  |  |
| 1. Taking advantage when they were too drunk or out of it to stop what was happening.. |  | |  | |  |  |
| 1. Threatening physical harm to them or someone close to them. |  | |  | |  |  |
| 1. Using force, like holding them down with my body weight, pinning their arms, or having a weapon. |  | |  | |  |  |
| 1. **I ATTEMPTED to (but did not) put in my penis, fingers or objects into a woman’s vagina when she did not agree to it by:** |  | | | | | |
| 1. Telling lies, threatening to end the relationship, threatening to spread rumors about them, making false promises, or verbally pressuring them after they said they didn’t want to. |  | |  | |  |  |
| 1. Showing displeasure, criticizing their sexuality or attractiveness, getting angry but not using physical force after they said they didn’t want to*.* |  | |  | |  |  |
| 1. Taking advantage when they were too drunk or out of it to stop what was happening. |  | |  | |  |  |
| 1. Threatening physical harm to them or someone close to them. |  | |  | |  |  |
| 1. Using force, like holding them down with my body weight, pinning their arms, or having a weapon. |  | |  | |  |  |
| 1. **I ATTEMPTED to (but did not) put my penis, fingers or objects into someone’s butt when they did not agree to it by:** |  | | | | | |
| 1. Telling lies, threatening to end the relationship, threatening to spread rumors about them, making false promises, or verbally pressuring them after they said they didn’t want to. |  | |  | |  |  |
| 1. Showing displeasure, criticizing their sexuality or attractive-ness, getting angry but not using physical force after they said they didn’t want to*.* |  | |  | |  |  |
| 1. Taking advantage when they were too drunk or out of it to stop what was happening. |  | |  | |  |  |
| 1. Threatening physical harm to them or someone close to them. |  | |  | |  |  |
| 1. Using force, like holding them down with my body weight, pinning their arms, or having a weapon. |  | |  | |  |  |
| Choices (if answer to any of #1-7)  1, zero times in the past 6 months  2, one time in the past 6 months  3, two times in the past 6 months  4, three or more times in the past 6 months | Skip  GO TO 9  GO TO 8  GO TO 8  GO TO 8 | | | | | |
| 1. What was the sex of the person or persons to whom you did any of the experiences that you mentioned? | 1, Female only  2, Male only  3, Both females and males | | | | | |
| 1. Have you ever raped someone? | 1, Yes  0, No | | | | | |

**Thank you for taking the time to complete this survey. Please return your tablet to the research assistant to collect your payment.**
